# Global, regional, and national trends in colorectal cancer from 2010 to 2021: an analysis of the global burden of disease study 2021

**DOI:** 10.1080/07853890.2025.2534098

**Published:** 2025-08-01

**Authors:** Gerun Chen, Jialin Wu, Sibo Huang, Zhenqi Gong, Huaiming Wang

**Affiliations:** a Department of Gastrointestinal Surgery, The First Affiliated Hospital of Shantou University Medical College, Shantou, China; b Shantou University Medical College, Shantou, China; c Department of Anatomical and Cellular Pathology, State Key Laboratory of Translational Oncology, Sir Y.K. Pao Cancer Center, Prince of Wales Hospital, The Chinese University of Hong Kong, Hong Kong, China; d Department of Interventional Radiology, The Third Affiliated Hospital of Sun Yat-sen University, Guangzhou, China; e Department of Radiology, The First Affiliated Hospital of Shenzhen University, Shenzhen University, Shenzhen Second People’s Hospital, Shenzhen, China

**Keywords:** Colorectal cancer, global burden of disease study, incidence, mortality, disability-adjusted life years

## Abstract

**Background:**

Colorectal cancer (CRC) is a major global health challenge due to its high incidence and mortality rates. Understanding its epidemiological trends is essential for informing prevention and intervention strategies.

**Methods:**

This study analyzed global, regional, and national CRC data from 2010 to 2021, focusing on age-standardized incidence, mortality, disability-adjusted life years (DALY), and associated risk factors. Data was sourced from the Global Burden of Disease (GBD) Study 2021 and stratified by the Sociodemographic Index (SDI).

**Results:**

Globally, CRC incidence increased by 35.91% from 1,614,410 cases in 2010 to 2,194,143 cases in 2021. The mortality rate decreased from 13.37 per 100,000 in 2010 to 12.40 per 100,000 in 2021. DALY increased by 23.94%, yet the age-standardized DALY rate (ASDR) decreased by 6.88%. Notably, high SDI regions showed a downward trend in incidence and mortality, while high-middle SDI regions experienced increasing incidence but decreasing mortality. In 2021, the highest incidence was in the Netherlands (69.80 per 100,000), and the highest mortality was in Uruguay (27.46 per 100,000). Dietary and metabolic risk factors significantly contributed to CRC burden across all SDI levels.

**Conclusion:**

The study reveals a complex epidemiological landscape of CRC, marked by rising incidence but declining mortality globally. These findings highlight the urgent need for targeted public health interventions, particularly in high-risk regions and younger populations, focusing on modifiable lifestyle and socioeconomic factors. Continuous monitoring and tailored prevention strategies are critical to reducing the global burden of CRC.

## HIGHLIGHTS

1. There are significant differences in the global burden of colorectal cancer across age, sex, and region.
2. The incidence, mortality, and disability-adjusted life years of colorectal cancer are higher in males than in females, with the highest proportion in the age group over 70 years old.
3. Dietary and lifestyle factors are the main contributors to the burden of colorectal cancer.

## Introduction

Colorectal cancer (CRC) has become a pressing global health issue, characterized by its high incidence and mortality rates. According to the Global Burden of Disease (GBD) Study 2021, CRC accounted for approximately 1.9 million new cases and over 935,000 deaths in 2020, making it the third most incidence cancer and the second leading cause of cancer-related mortality worldwide [1]. The burden of CRC is projected to escalate further, with estimates suggesting a 60% increase in cases by 2030 [2], driven by demographic changes, lifestyle factors, and environmental influences.

The rising incidence of CRC is closely linked to various modifiable risk factors, including unhealthy diet, alcohol intake, smoking, obesity, lifestyle choices and insufficient physical activity. A meta-analysis reports that for every 100 g/day increase in consumption of red and processed meat, the risk of CRC increases by 12%, and for every 10 g/day increase in ethanol intake derived from alcoholic beverages, the risk of CRC increases by 7% [3]. Beyond dietary factors, a sedentary lifestyle and obesity further contribute to an increased risk of CRC^4^. Furthermore, age remains a key determinant, with individuals aged over 50 being at the greatest risk [4]. Additionally, Sales et al. have demonstrated that psychosocial factors, including stress and mental health status, may impact patients with CRC, highlighting the multifaceted nature of this disease [5].

The GBD Study provides valuable insights into the epidemiological trends of CRC, underscoring the importance of early detection and intervention. Early diagnosis significantly improves prognosis; however, awareness of CRC risk factors remains alarmingly low in many populations. For instance, studies conducted in various countries have reported that a substantial proportion of individuals are unaware of the key risk factors associated with CRC, which hampers early detection efforts [6]. The study by Sung et al. reveals an increasing incidence of early-onset CRC in 27 of 50 countries/regions with the most notable annual rises in New Zealand Chile and Puerto Rico at 4.0% 4.0% and 3.8% respectively and in 14 countries/regions including the USA the incidence is going up among the young while it stays stable in those aged 50 and above [7]. This trend underscores the urgent need to enhance awareness and prevention efforts in younger populations. This gap in knowledge emphasizes the necessity for educational campaigns aimed at increasing public awareness and understanding of CRC risk factors. Furthermore, the molecular heterogeneity of CRC presents challenges in treatment and management. Variations in tumor biology can influence patient outcomes and responses to therapy, necessitating personalized treatment approaches [8]. Wang et al. have identified specific genetic markers and molecular subtypes associated with CRC, which may help to tailor interventions and enhance the accuracy of prognosis [9].

The burden of CRC is a pressing global health issue, driven by a complex interplay of lifestyle, demographic, and molecular factors. As the incidence and mortality rates continue to rise, it is imperative to enhance public awareness, promote healthy lifestyle choices, and invest in research aimed at understanding the underlying mechanisms of CRC. The findings from the GBD Study 2021 provide a critical foundation for future efforts to combat this disease and improve outcomes for affected individuals. Based on the latest GBD 2021 data, our study aims to update and assess global, regional, and national CRC trends over the past 12 years by analyzing incidence, mortality, disability-adjusted life years (DALY), and associated risk factors from 2010 to 2021.

## Methods

### Data sources

The Global Burden of Disease, Injuries, and Risk Factors Study (GBD) constitutes a comprehensive and ongoing global research collaboration. It systematically quantifies health loss attributabl^11^e to major diseases, injuries, and risk factors worldwide. GBD integrates diverse epidemiological data sources, including vital registration systems, disease registries, surveys, and scientific literature, applying standardized statistical models and estimation methods to yield comparable estimates of health metrics. The GBD 2021, which provides authoritative estimates spanning 204 countries and territories across 7 super-regions through 2021, serves as the primary data source for this CRC analysis [10]. This study encompasses comprehensive data on incidence, mortality, and DALY, particularly the age-standardized incidence rate (ASIR), age-standardized mortality rate (ASMR), and age-standardized DALY rate (ASDR) for CRC, which are derived from the 2021 GBD study. For clarity, crude rates represent the actual observed rates within the specific population age structure. In contrast, age-standardized rates (ASIR, ASMR, ASDR) are calculated by applying the age-specific rates of the study population to a standard population structure, enabling valid comparisons of disease burden across different populations or over time that are not confounded by differences in age distribution. Given the focus of this analysis on comparisons across diverse geographical locations and over time, the GBD study primarily employs and reports age-standardized rates, which are the metrics utilized in this CRC analysis. The dataset obtained includes various dimensions, such as gender, age, and geographical location. However, analyses related to race and ethnicity were not performed due to the absence of such parameters in the GBD database. The research methodology employed by the GBD has been thoroughly documented in the existing literature [10,11].

### Sociodemographic Index

The Sociodemographic Index (SDI) is a multifaceted metric that quantifies the socio-economic development of a country or region. This index integrates various dimensions, including economic structure and size, educational attainment, living standards, and the extent of social welfare and security. The SDI values range from 0 to 1, with higher values signifying a greater level of socio-economic development. The classification of regions based on SDI is divided into five categories: low (0 to 0.46), low-middle (0.46 to 0.60), middle (0.61 to 0.69), high-middle (0.70 to 0.81), and high (0.81 to 1). This stratification enables a nuanced examination of how socio-economic indices and geographic disparities influence the burden of disease.

### Statistical analysis

Incidence, mortality and DALY, along with their corresponding 95% uncertainty interval (UI), were calculated per 100,000 population based on data from the GBD database. The average estimated annual percentage changes (EAPC) and its Confidence intervals (CI) were derived using a log-transformed linear regression model to analyze temporal trends in CRC incidence, mortality and DALY from 2010 to 2021. The EAPC, a standard metric quantifying temporal trends in age-standardized rates, is derived from the log-linear model ln(y) = α + βx + ε, where y denotes age-standardized incidence, α is the intercept, β is the slope coefficient, x denotes calendar year, and ε is a normally distributed error term. Computed as (exp^ᵝ − 1) × 100%, EAPC trends are clinically interpreted as: increasing when the lower 95% CI > 0, decreasing when the upper 95% CI < 0, or stable when the CI spans zero. Fitted curves were employed to examine the relationship between disease burden indicators and the SDI. Utilizing the methodology established by Das Gupta, we decomposed the changes in CRC incidence and mortality across regions from 2010 to 2021, attributing these changes to factors such as epidemiological shifts, population growth, and aging [12]. The observed net change in the total number of cases corresponded to the cumulative effects of these two factors. A two-sided p-value less than 0.05 was considered significant. Data cleaning, computation, and visualization were conducted using JD_GBDR (version 2.36.1, Jingding Medical Technology Co., Ltd.) and R software (version 4.4.2) in this study.

### Ethical approval and consent to participate

The institutional review board of the First Affiliated Hospital of Shantou University Medical College in Guangdong Province, China, determined that the study did not need approval because it used publicly available data.

## Result

### Global burden trends

#### Incidence

**Figure 1.**
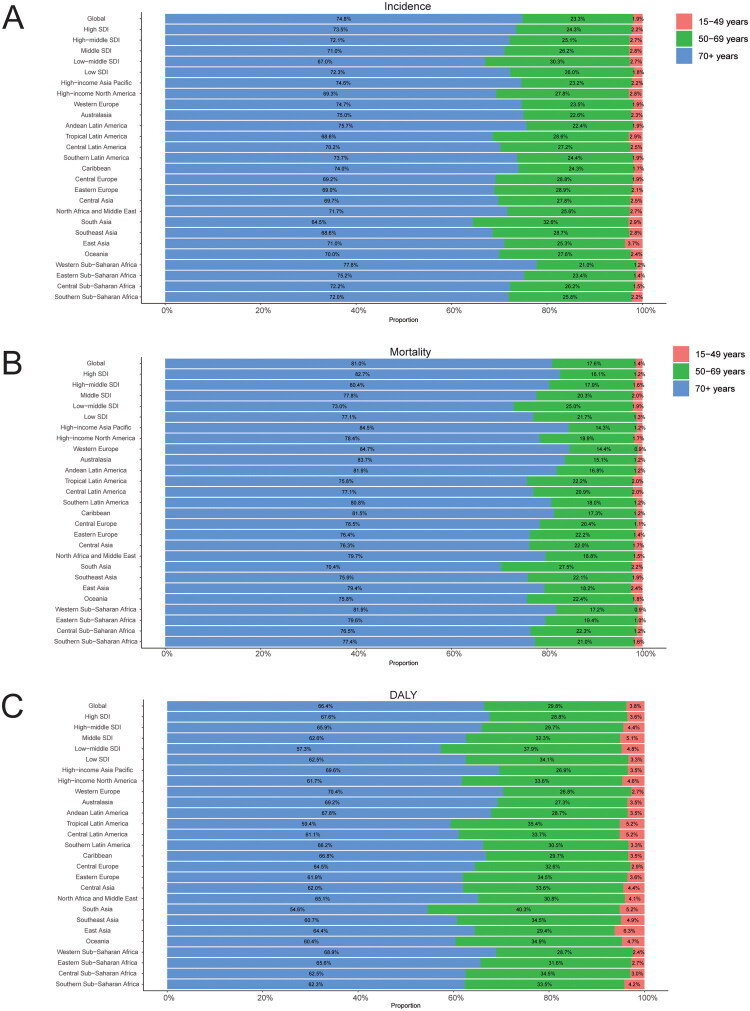
Age-related percentages of colorectal cancer incidence (A), mortality (B), and DALY (C) in 2021.

**Figure 2.**
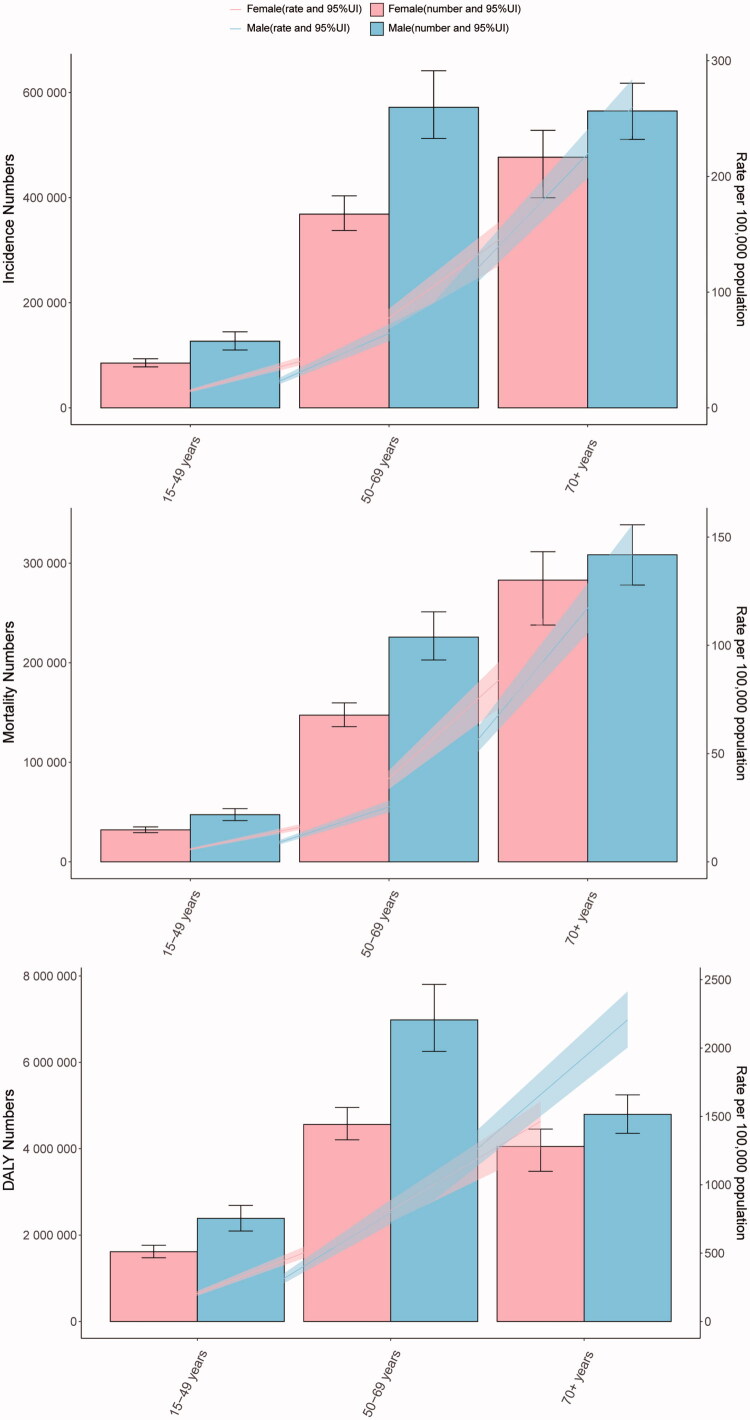
Age-related and sex-related burden of colorectal cancer in 2021 of incidence (A), mortality (B), and DALY (C).

In 2010, the global incidence of CRC was reported to be 1,614,410 (95% UI 1,526,437 to 1,671,789) cases (Table S1). By 2021, this figure had risen to 2,194,143 (95% UI 2,001,271 to 2,359,390) cases, representing a 35.91% increase. However, the incidence rates for 2021 were comparable to those of 2010, recorded at 25.61 (95% UI 23.32 to 27.52) per 100,000 and 25.61 (95% UI 24.06 to 26.54), respectively, indicating no significant difference overall (Table 1). The EAPC was calculated at 1.67 (95% CI 1.61 to 1.74). Notably, the incidence rates of CRC increased in both the 15–49 years and 50–69 years age groups, with an increase of 8.58 and 3.39, respectively. Conversely, a decrease was observed in the age group over 70 years, with a rate of −0.77 (Table S2). The highest incidence of CRC was found in individuals aged over 70, accounting for 74.8% of all cases reported in 2021, with a corresponding incidence rate of 210.72 (95% UI 187.94 to 227.06) per 100,000 (Figure 1A and Table S2). In contrast, the 15–49 years age group consistently exhibited the lowest incidence rate, comprising only 1.9% of all CRC cases that year, with an incidence rate of 5.37 (95% UI 4.91 to 5.86) per 100,000. Regarding gender differences, male incidence rates were higher than those of females across all age groups, with the most pronounced disparity observed in individuals aged over 70 (Figure 2A).

**Table 1.** Age-standardized incidence, mortality, and DALY rates for colorectal cancer in 2010 and 2021 and their temporal trends from 2010 and 2021.

Locations	Age-standardized incidence rate per 100,000 population			Age-standardized mortality rate per 100,000 population			Age-standardized DALY rate per 100,000 population		
	2010 No. (95%UI)	2021 No. (95%UI)	EAPC No. (95%CI)	2010 No. (95%UI)	2021 No. (95%UI)	EAPC No. (95%CI)	2010 No. (95%UI)	2021 No. (95%UI)	EAPC No. (95%CI)
Global	25.61 (24.06,26.54)	25.61 (23.32,27.52)	1.67 (1.61,1.74)	13.37 (12.40,13.91)	12.40 (11.24,13.31)	1.10 (1.01,1.19)	25.61 (24.06,26.54)	25.61 (24.06,26.54)	25.61 (24.06,26.54)
SDI									
High SDI	44.01 (41.09,45.60)	40.53 (37.44,42.45)	0.74 (0.62,0.86)	17.04 (15.58,17.81)	15.02 (13.58,15.93)	0.66 (0.58,0.75)	44.01 (41.09,45.60)	44.01 (41.09,45.60)	44.01 (41.09,45.60)
High-middle SDI	31.69 (29.88,33.15)	34.00 (30.33,37.95)	2.58 (2.49,2.67)	17.05 (16.04,17.80)	15.71 (14.14,17.25)	1.33 (1.24,1.42)	31.69 (29.88,33.15)	31.69 (29.88,33.15)	31.69 (29.88,33.15)
Middle SDI	16.40 (15.42,17.35)	19.55 (17.14,22.04)	4.02 (3.83,4.21)	10.61 (9.97,11.22)	10.65 (9.42,11.84)	2.55 (2.40,2.71)	16.40 (15.42,17.35)	16.40 (15.42,17.35)	16.40 (15.42,17.35)
Low-middle SDI	7.34 (6.84,7.89)	8.20 (7.51,8.96)	2.73 (2.56,2.89)	6.28 (5.85,6.74)	6.55 (6.02,7.17)	2.13 (1.95,2.30)	7.34 (6.84,7.89)	7.34 (6.84,7.89)	7.34 (6.84,7.89)
Low SDI	6.78 (6.14,7.47)	7.39 (6.65,8.19)	1.40 (1.30,1.49)	6.53 (5.92,7.18)	6.88 (6.18,7.62)	0.99 (0.89,1.08)	6.78 (6.14,7.47)	6.78 (6.14,7.47)	6.78 (6.14,7.47)
Regions									
Andean Latin America	13.03 (11.53,14.84)	14.39 (11.40,17.89)	2.93 (2.17,3.69)	10.29 (9.03,11.65)	10.00 (7.98,12.18)	1.74 (1.03,2.45)	13.03 (11.53,14.84)	13.03 (11.53,14.84)	13.03 (11.53,14.84)
Australasia	49.66 (44.87,53.70)	43.97 (38.91,49.55)	0.12 (-0.24,0.48)	16.96 (15.07,18.42)	14.62 (12.77,16.52)	0.10 (-0.27,0.47)	49.66 (44.87,53.70)	49.66 (44.87,53.70)	49.66 (44.87,53.70)
Caribbean	31.81 (29.64,33.82)	34.33 (29.87,38.86)	2.46 (2.24,2.69)	14.47 (13.39,15.53)	14.57 (12.72,16.60)	1.85 (1.67,2.04)	31.81 (29.64,33.82)	31.81 (29.64,33.82)	31.81 (29.64,33.82)
Central Asia	10.88 (10.15,11.69)	10.82 (9.69,11.90)	1.28 (0.98,1.59)	8.52 (7.93,9.16)	7.87 (7.06,8.66)	0.33 (0.05,0.61)	10.88 (10.15,11.69)	10.88 (10.15,11.69)	10.88 (10.15,11.69)
Central Europe	38.40 (36.69,39.87)	38.82 (35.71,41.96)	1.73 (1.58,1.89)	23.84 (22.67,24.80)	22.59 (20.81,24.28)	1.37 (1.24,1.50)	38.40 (36.69,39.87)	38.40 (36.69,39.87)	38.40 (36.69,39.87)
Central Latin America	13.91 (13.23,14.48)	17.74 (15.76,19.81)	4.90 (4.74,5.06)	8.18 (7.69,8.52)	9.30 (8.26,10.35)	3.87 (3.70,4.04)	13.91 (13.23,14.48)	13.91 (13.23,14.48)	13.91 (13.23,14.48)
Central Sub-Saharan Africa	7.03 (5.54,8.62)	7.87 (6.09,10.54)	1.64 (1.60,1.68)	6.91 (5.41,8.62)	7.45 (5.77,10.14)	1.18 (1.13,1.22)	7.03 (5.54,8.62)	7.03 (5.54,8.62)	7.03 (5.54,8.62)
East Asia	25.89 (23.60,28.41)	31.60 (25.90,37.85)	4.51 (4.26,4.77)	14.20 (12.96,15.43)	13.78 (11.33,16.35)	2.63 (2.36,2.91)	25.89 (23.60,28.41)	25.89 (23.60,28.41)	25.89 (23.60,28.41)
Eastern Europe	29.84 (28.74,30.75)	32.11 (29.59,34.73)	2.10 (1.90,2.30)	18.93 (18.15,19.51)	18.06 (16.58,19.56)	1.00 (0.81,1.19)	29.84 (28.74,30.75)	29.84 (28.74,30.75)	29.84 (28.74,30.75)
Eastern Sub-Saharan Africa	10.31 (9.43,11.26)	11.23 (9.84,12.77)	1.69 (1.49,1.89)	10.17 (9.32,11.11)	10.76 (9.41,12.15)	1.33 (1.17,1.49)	10.31 (9.43,11.26)	10.31 (9.43,11.26)	10.31 (9.43,11.26)
High-income Asia Pacific	45.68 (41.88,48.07)	44.89 (40.20,47.85)	1.87 (1.55,2.19)	16.22 (14.44,17.19)	15.00 (13.03,16.09)	2.11 (1.91,2.30)	45.68 (41.88,48.07)	45.68 (41.88,48.07)	45.68 (41.88,48.07)
High-income North America	43.71 (40.70,45.50)	38.75 (36.14,40.48)	0.18 (0.03,0.34)	14.92 (13.66,15.63)	12.96 (11.89,13.65)	0.09 (-0.03,0.20)	43.71 (40.70,45.50)	43.71 (40.70,45.50)	43.71 (40.70,45.50)
North Africa and Middle East	12.75 (11.83,13.76)	14.43 (12.67,16.35)	2.93 (2.73,3.13)	8.80 (8.08,9.53)	8.95 (7.85,10.08)	1.78 (1.53,2.03)	12.75 (11.83,13.76)	12.75 (11.83,13.76)	12.75 (11.83,13.76)
Oceania	6.30 (5.56,7.04)	6.44 (5.55,7.42)	1.25 (1.10,1.41)	5.54 (4.90,6.22)	5.58 (4.80,6.42)	1.04 (0.88,1.19)	6.30 (5.56,7.04)	6.30 (5.56,7.04)	6.30 (5.56,7.04)
South Asia	4.86 (4.50,5.18)	5.65 (5.08,6.30)	3.44 (3.17,3.70)	4.31 (3.97,4.61)	4.63 (4.17,5.16)	2.72 (2.47,2.97)	4.86 (4.50,5.18)	4.86 (4.50,5.18)	4.86 (4.50,5.18)
Southeast Asia	15.66 (14.06,17.14)	17.70 (15.41,19.89)	3.43 (3.29,3.58)	12.29 (10.95,13.46)	12.74 (11.12,14.26)	2.60 (2.47,2.73)	15.66 (14.06,17.14)	15.66 (14.06,17.14)	15.66 (14.06,17.14)
Southern Latin America	29.98 (27.35,32.80)	28.32 (25.10,31.53)	0.94 (0.52,1.36)	21.12 (19.25,23.04)	18.13 (16.12,20.25)	0.08 (-0.36,0.53)	29.98 (27.35,32.80)	29.98 (27.35,32.80)	29.98 (27.35,32.80)
Southern Sub-Saharan Africa	13.55 (12.74,14.33)	13.45 (12.16,14.83)	1.41 (1.18,1.64)	12.29 (11.53,13.01)	11.47 (10.37,12.61)	0.77 (0.54,0.99)	13.55 (12.74,14.33)	13.55 (12.74,14.33)	13.55 (12.74,14.33)
Tropical Latin America	15.45 (14.41,16.28)	17.17 (15.82,18.31)	3.37 (3.21,3.54)	11.40 (10.56,12.04)	11.58 (10.59,12.34)	2.68 (2.50,2.85)	15.45 (14.41,16.28)	15.45 (14.41,16.28)	15.45 (14.41,16.28)
Western Europe	45.45 (42.13,47.40)	40.54 (37.17,43.07)	0.24 (0.03,0.46)	17.80 (16.08,18.72)	15.11 (13.53,16.25)	0.18 (0.02,0.34)	45.45 (42.13,47.40)	45.45 (42.13,47.40)	45.45 (42.13,47.40)
Western Sub-Saharan Africa	5.96 (5.24,6.63)	6.29 (5.36,7.30)	0.52 (0.40,0.64)	5.84 (5.17,6.46)	5.98 (5.12,6.88)	0.15 (-0.01,0.32)	5.96 (5.24,6.63)	5.96 (5.24,6.63)	5.96 (5.24,6.63)

DALY: Disability-adjusted life year, CRC: colorectal cancer, UI: Uncertainty interval, SDI: Sociodemographic Index.

#### Mortality

In 2010, the global mortality due to CRC was reported at 820,414 (95% UI 767,047 to 851,834) deaths (Table S1). By 2021, this figure had risen to 1,044,072 (95% UI 950,187 to 1,120,169) deaths, representing an overall increase of 27.26%. However, the mortality rate decreased from 13.37 (95% UI 12.40 to 13.91) per 100,000 in 2010 to 12.40 (95% UI 11.24 to 13.31) per 100,000 in 2021 (Table 1). The EAPC was calculated at 1.10 (95% UI 1.01 to 1.19). It is noteworthy that the incidence of CRC declined across all age brackets, with the most significant decrease observed in those aged over 70 years (−6.09 95% UI −10.82 to −1.34), followed by the 50–69 years age group (−4.38 95% UI −11.20 to 3.42), and the least decrease in the 15–49 years age group (−2.03 95% UI −9.78 to 7.07) (Table S2). CRC mortality was highest in individuals aged over 70, accounting for 81.0% of all deaths due to CRC in 2021, with a corresponding mortality rate of 119.64 (95% UI 106.19 to 128.90) per 100,000 (Figure 1B and Table S2). In contrast, the 15–49 years age group consistently exhibited the lowest mortality rate, comprising only 1.4% of all CRC deaths that year, with a mortality rate of 2.01 (95% UI 1.84 to 2.19) per 100,000. Regarding gender differences, male mortality rates were higher than those of females across all age brackets, with the most pronounced disparity observed in individuals aged over 70 (Figure 2B).

#### DALY

In 2010, the global burden of CRC, DALY was 19,687,652 (95% UI 18,818,729 to 20,373,871) (Table S1). By 2021, this figure had increased to 24,401,100 (95% UI 22,689,369 to 26,161,518), representing an overall increase of 23.94%. However, the ASDR per 100,000 population decreased from 304.16 (95% UI 289.85 to 315.08) in 2010 to 283.24 (95% UI 263.11 to 303.33) in 2021, indicating an overall reduction of 6.88% (Table 1). DALY values declined across all age brackets, with the most significant reduction observed in individuals aged over 70 years (−7.41 95% UI −12.35 to −2.43), followed by those aged 50–69 years (−4.96 95% UI −11.84 to 2.88), and the least reduction noted in the 15–49 years age group (-1.88 95% UI −9.71 to 7.20) (Table S2). In terms of age groups, individuals aged over 70 years exhibited the highest rate of DALY associated with CRC, accounting for 66.4% of all DALY reported in 2021, with a corresponding rate of 1,790.14 (95% UI 1,602.69 to 1,924.17) per 100,000 (Figure 1C and Table S2). In contrast, the 15–49 years age group consistently demonstrated the lowest DALY rate, comprising only 3.8% of all CRC-related DALY in 2021, with a DALY rate of 101.37 (95% UI 92.85 to 110.18) per 100,000. Furthermore, there were notable gender differences in DALY counts, with males exhibiting higher DALY values than females across all age brackets, particularly pronounced in individuals aged over 70 (Figure 2C).

#### Regional trends by SDI

**Figure 3.**
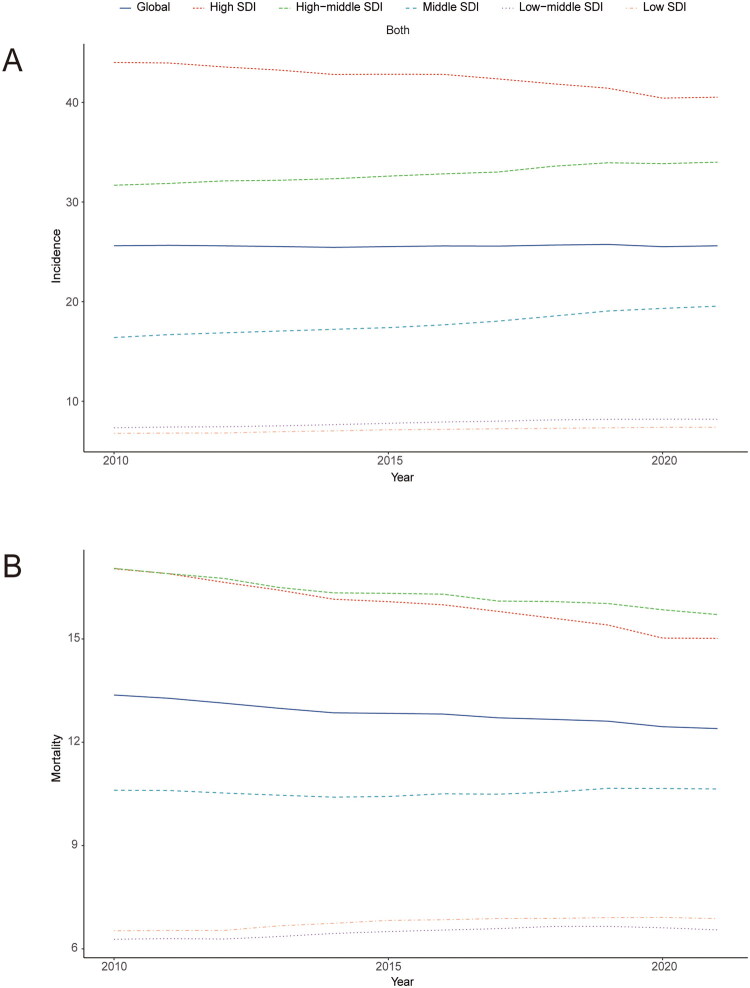
Temporal trends in incidence (A) and mortality (B) of colorectal cancer across different Sociodemographic Index (SDI) levels from 2010 to 2021.

**Figure 4.**
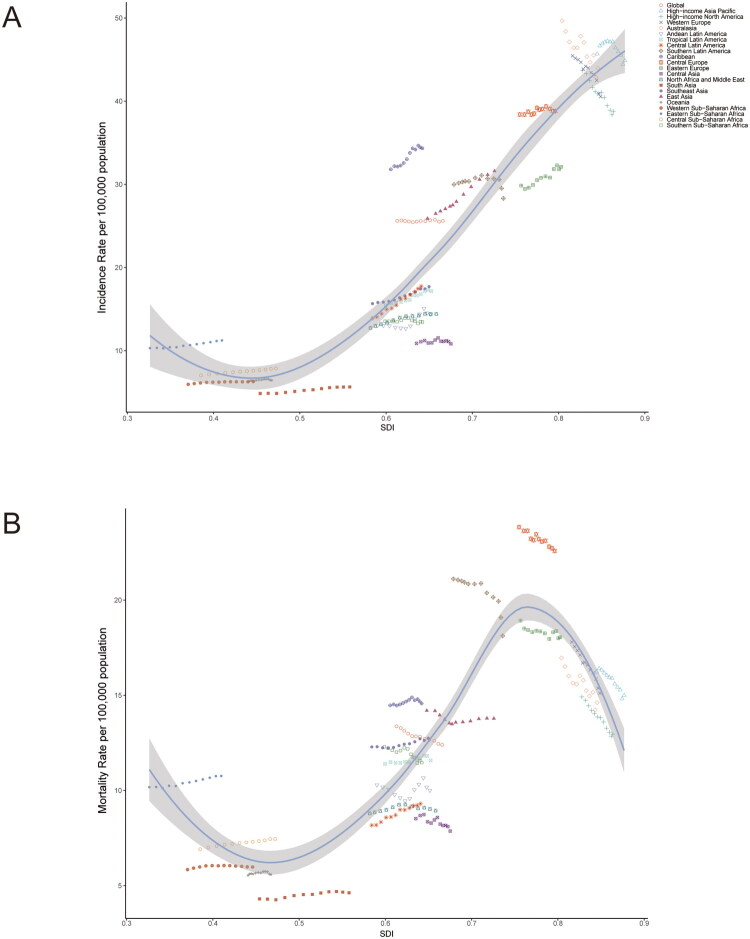
Association between incidence (A), mortality (B) rates of colorectal cancer and regional Sociodemographic Index (SDI), 2010–2021.

Between 2010 and 2021, the incidence and mortality rates of CRC exhibited divergent trends across regions with varying SDI levels. Notably, high SDI regions, despite having elevated incidence and mortality rates, demonstrated a downward trajectory in both metrics. In contrast, regions categorized as having high-middle SDI levels also reported high incidence and mortality rates; however, while the incidence rate showed an increasing trend, the mortality rate exhibited a decline. The remaining three SDI categories displayed relatively stable trends in both incidence and mortality rates, with the exception of the middle SDI category, where an upward trend in incidence was observed (Figure 3). Figure 4 illustrates the long-term trends of ASMR and ASIR for CRC, stratified by geographical regions across five quintile categories of SDI from 2010 to 2021. Furthermore, it presents predicted rates derived from a comprehensive global SDI measurement. A significant disparity in the impact of SDI across different regions is evident. The incidence rate initially decreases and then increases with rising SDI, reaching a minimum at an SDI of 0.45. Conversely, the mortality rate follows a more complex trajectory, initially declining, then increasing, and subsequently decreasing again, with a minimum at an SDI of 0.46 and a peak at 0.77 (Figure 4). These observations suggest that while the incidence of CRC is likely to rise with increasing SDI, the mortality rate is anticipated to decrease as SDI increases.

### National trends

#### Incidence

**Figure 5.**
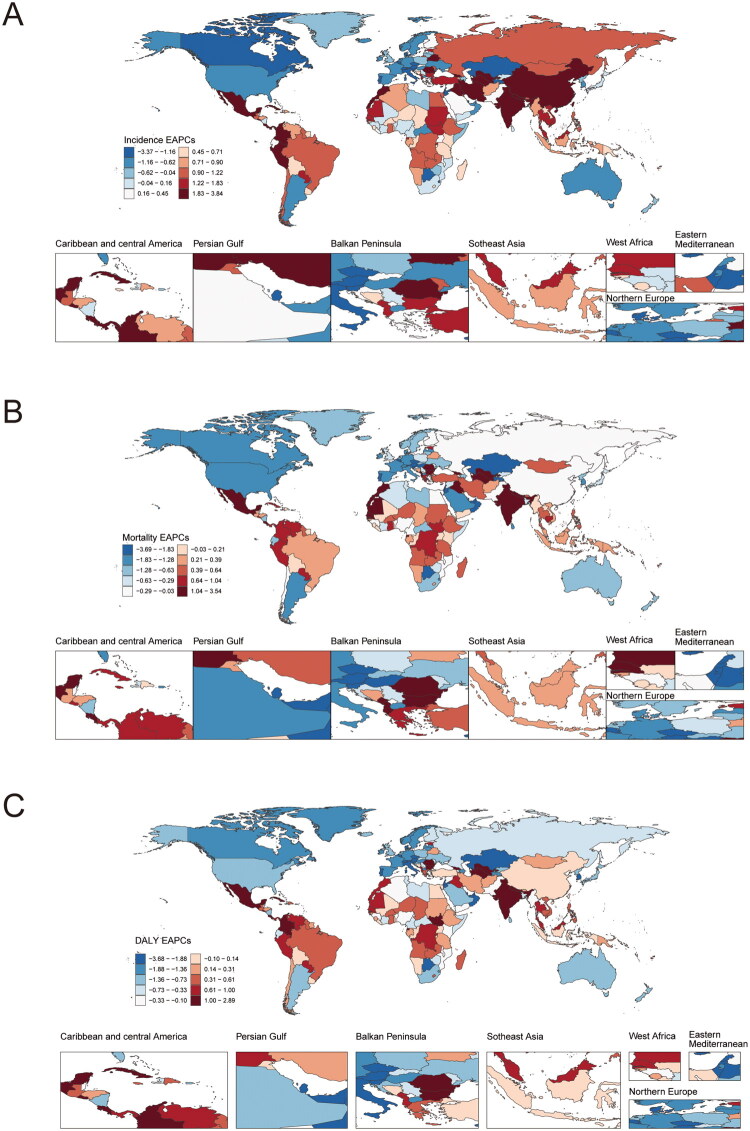
The EAPC of colorectal cancer incidence (A), mortality (B), and DALY (C) across 204 countries and territories between 2010 and 2021.

In 2021, the Netherlands reported the highest CRC incidence globally, with an age-standardized rate of 69.80 (95% UI 62.21 to 76.79) cases per 100,000 population (Table S3). Conversely, The Gambia exhibited the lowest incidence rate, at 3.31 (95% UI 2.54 to 4.27) cases per 100,000 population. From 2010 to 2021, Cabo Verde experienced the most significant increase in CRC incidence, with an EAPC of 3.80% (95% CI 3.06% to 4.54%) (Figure 5A and Table S4). In contrast, San Marino demonstrated the largest decrease in incidence during the same period, with an EAPC of −3.34% (95% CI −5.63% to −0.99%). The global CRC incidence rate in 2021 was recorded at 25.61 (95% UI 23.32 to 27.52) cases per 100,000 population, which was higher than the incidence rates observed in 133 out of 204 countries, while being lower than that in 71 countries.

#### Mortality

In 2021, Uruguay exhibited the highest CRC mortality rate globally, with an incidence of 27.46 (95% UI 24.25 to 30.91) per 100,000 individuals (Table S3). Conversely, The Gambia reported the lowest mortality rate, at 3.05 (95% UI 2.34 to 3.93) per 100,000 individuals. Between 2010 and 2021, Cabo Verde experienced the most significant increase in mortality rate, with an EAPC of 3.50% (95% CI 2.67% to 4.34%) (Figure 5B and Table S4). In contrast, Qatar demonstrated the most substantial decrease in mortality rate, with an EAPC of −3.65% (95% CI −4.93% to −2.35%). The global CRC mortality rate in 2021 was recorded at 12.40 (95% UI 11.24 to 13.31) per 100,000 individuals, surpassing the mortality rates of 109 out of 204 countries, while remaining lower than that of 95 countries.

#### DALY

In 2021, Hungary had the highest ASDR for CRC (614.96 per 100,000 population; 95% UI 519.37 to 736.20), whereas Gambia had the lowest (70.75 per 100,000 population; 95% UI 53.17 to 92.31) (Figure 5C and Table S3). Between 2010 and 2021, Cabo Verde had the largest increase in ASDR (EAPC = 2.86%; 95% CI 2.11 to 3.61), and Qatar had the largest decrease in ASDR (EAPC = −3.64%; 95% CI −4.40 to −2.86) (Figure 5C and Table S4). In 2021, the global ASDR for CRC was 283.24 (95% UI 263.11 to 303.33) per 100,000 population, higher than that of 107 countries out of 204, and lower than that of 97 countries (Table S3).

#### CRC risk factors

**Figure 6.**
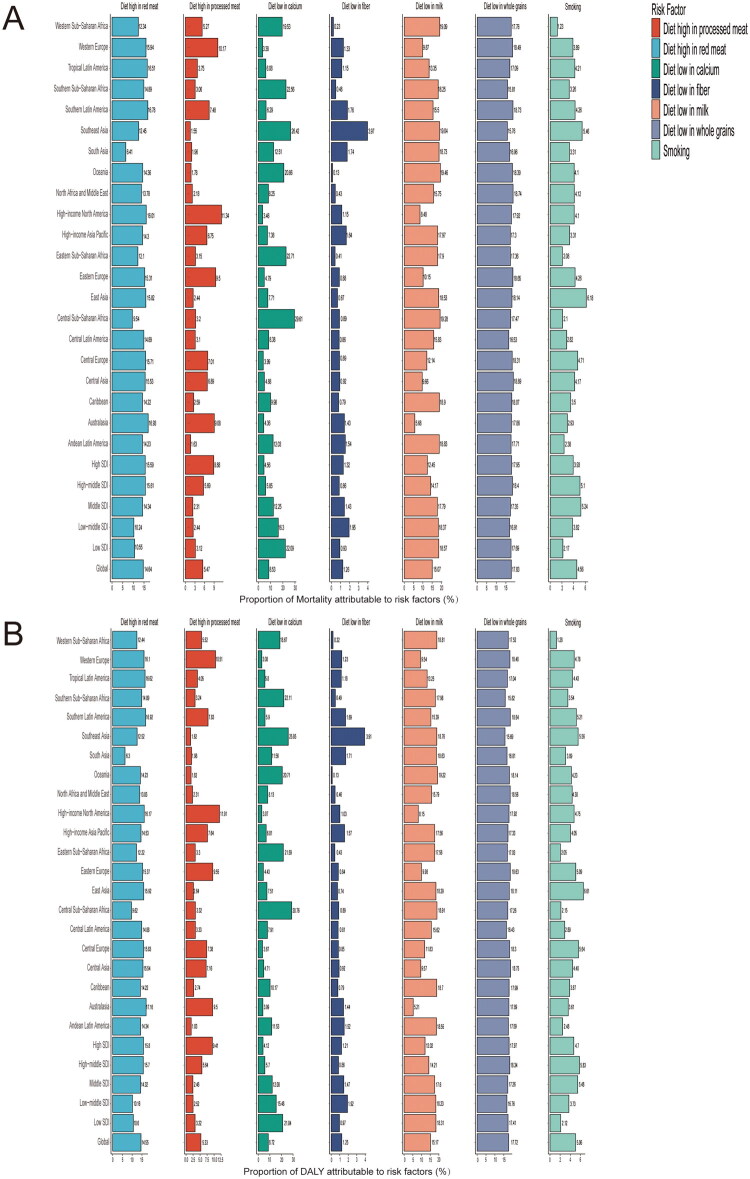
Proportion of colorectal cancer mortality (A) and DALY (B) attributable to risk factors across different Sociodemographic Index (SDI) levels.

Globally, dietary factors such as high intake of red and processed meat and low intake of milk and grains significantly impact the CRC burden across all SDI levels. In terms of the proportion of mortality attributable to risk factors, low grain diet, low milk diet, and high red meat intake account for 17.83%, 15.07%, and 14.64% (Figure 6A), respectively. Regarding the proportion of DALY, they account for 17.72%, 15.17%, and 14.55% (Figure 6B), respectively. In addition to dietary factors, smoking is also a significant contributor. Globally, smoking accounts for 4.56% of mortality and 5.06% of DALY. In age-specific analysis, the impact of dietary factors is almost the same across different age groups, with the highest proportion of low grain diet. In individuals aged 70 and above, it contributes 17.92% (Figure S1A) to mortality and 17.92% (Figure S1B) to DALY; in those aged 50–69, the contributions are 17.8% and 17.8%, respectively; and in those aged 15–49, the contributions are 17.26% and 17.04%, respectively. However, the impact of smoking varies across different age groups. In individuals aged 50–69, it has the highest proportion, contributing 6.04% to mortality and 6.1% to DALY, differing from other age groups. In those aged 70 and above, the contributions are 3.59% and 3.91%, and in those aged 15–49, the contributions are 4.78% and 4.6%.

## Discussion

Our analysis offers distinct methodological advantages over prior GBD-based studies. Notably, whereas Wang et al. examined CRC trends from 1990 to 2021, we focused on the period 2010–2021 to capture substantial shifts in the global CRC burden during the past decade [13]. This time frame was selected because CRC data prior to 2010 in the GBD repository exhibit sparser coverage and comparatively limited reliability, enabling our study to leverage higher-quality contemporary evidence. Over the past 12 years, there has been a marked increase in the number of new cases, deaths, and DALY attributable to CRC worldwide. Although these absolute figures have been rising, the ASIR has remained relatively stable overall, while the ASMR and ASDR have shown a declining trend. The age group over 70 years old accounted for the highest proportion in ASIR, ASMR, and ASDR, and in all age groups, the ASIR, ASMR, and ASDR of males were higher than those of females. Despite the continuous decline in global ASIR and ASDR values, population aging, lifestyle changes, and population growth may lead to a higher absolute incidence and mortality of CRC. Despite significant efforts in CRC prevention and control, the substantial increase in absolute numbers remains a major concern. Our research findings highlight the complex interplay between demographic, lifestyle, and socioeconomic factors driving the incidence, mortality, and DALY of CRC. These trends emphasize the urgent need for targeted public health interventions and further research to mitigate the impact of CRC on global health.

Regional disparities in CRC incidence highlight the impact of socioeconomic development on the disease burden. Although the incidence in high SDI regions, such as Australia and New Zealand, is significantly higher than that in low and medium SDI regions, it is showing a downward trend, while middle and high SDI regions are showing an upward trend. This worrying trend is instigated by a range of elements, including swift population growth, an ageing population, and alterations in lifestyle. The fastest population growth in low- and middle-income countries inevitably leads to an increased cancer burden [14]. In contrast, the stable population and low fertility rate in high SDI countries can have an impact on disease incidence. A stable population implies a relatively stable demographic structure, and the incidence of diseases may also tend to stabilize. The low fertility rate leads to a reduction in the number of children and young individuals, which may decrease the incidence of some childhood and youth-related diseases. However, with the rapid aging of the national population, there is an inevitable increase in the incidence of CRC [15]. Arima et al. have showed that a Western-style diet is associated with a higher incidence of CRC containing a large amount of pks + E coli [16].This may be due to the adoption of a Western lifestyle without the implementation of effective preventive measures [17]. Therefore, alterations in dietary habits in developing countries in recent years have been another factor contributing to the increased incidence of CRC [18]. In high SDI regions, the decline in the ASIR is closely associated with advanced medical infrastructure, abundant medical resources, and a high level of public health awareness [19,20]. These advantages have led to more widespread application and promotion of cancer screening and early-diagnosis technologies. For example, the popularity of colonoscopy has significantly increased in these areas, enabling the detection of CRC at an early or even precancerous stage, thus greatly improving the cure rate and reducing the incidence [21]. Regarding the incidence of CRC in different age groups, our research results show that the incidence in people over 70 years old is the highest, which emphasizes the need for targeted screening and prevention strategies in this high-risk group. However, the increase in incidence in the younger age group (15–49 years old) is also a cause for concern, indicating that CRC is no longer limited to the elderly population. This change may be attributed to the adoption of unhealthy lifestyle habits in the younger population, such as sedentary behavior and poor dietary choices [22].

The trends in CRC mortality present a more complex picture. Although the overall global mortality rate has declined, the number of deaths has increased. The highest mortality rate was observed in individuals aged over 70 years, reflecting the cumulative effect of risk factors over time and the limited treatment effectiveness in this age group. The decline in mortality in high SDI areas indicates that advances in medical technology, coupled with the efforts of clinicians, have significantly improved the prognosis of CRC patients [23]. For instance, in high-income countries, the use of advanced imaging technologies and targeted therapies has improved the prognosis of CRC patients [24]. However, the persistently high mortality rates in regions with low and middle SDI levels highlight the disparities in healthcare access in these areas, as well as the urgent need for capacity-building. The notable decline in mortality in countries like Qatar and San Marino illustrates the potential for other regions to achieve similar improvements through targeted interventions and investments in medical infrastructure [25]. To enhance the overall prognosis of cancer in the population, it is essential to rely on screening programs with wide coverage and high quality, which can help reduce the burden of CRC on the population. Despite the high efficacy of CRC screening in detecting early-stage malignancies and precancerous conditions, substantial disparities exist in screening practices and participation rates among various regions. Data show that among four countries with nationwide CRC screening programs, three countries had a fecal test usage rate of over 50% (UK: 59%, Slovenia: 56%, France: 51%); Croatia was an exception, with only 22% [26]. In addition, various screening methods are employed across different regions, including faecal occult blood testing, serum tumour marker analysis, colonoscopy, and sigmoidoscopy. Kahi et al. have shown that colonoscopy can reduce CRC mortality [27]. However, different countries have implemented diverse screening strategies for CRC. The United Kingdom, Canada, Australia, Japan, Taiwan, and South Korea primarily rely on fecal immunochemical testing as their main screening method. In contrast, the United States, Germany, Poland, Israel, and Abu Dhabi predominantly utilize colonoscopy for CRC screening. Additionally, flexible sigmoidoscopy and CT colonography are also employed in some countries and regions as part of their screening programs [28]. Wooldrage et al. have demonstrated that the UK’s CRC screening program, which involved flexible sigmoidoscopy, led to a 25% reduction in CRC mortality and a 24% decrease in incidence [29]. Therefore, further comparisons of screening programs across different regions will help to reveal how these factors affect the ASIR and the ASMR. Meanwhile, countries can reduce the disease burden of CRC by establishing more comprehensive health policies and healthcare systems to improve relevant medical services.

The evaluation of DALY provides a comprehensive indicator for gauging the overall burden of CRC, considering both the years of life lost as a result of early death and the years lived while affected by the disease. The decline in DALY across all age groups is encouraging, indicating that global efforts to reduce the impact of CRC are having some effect. However, the persistent high DALY rates in the elderly group and high SDI areas indicate that further improvement is needed. The substantial contribution of dietary and metabolic risk factors to DALY underscores the critical need for public health interventions targeting these modifiable factors. Our study confirms that dietary factors including high consumption of processed meat, a low-fiber diet, and low calcium intake, and metabolic risk factors including a high body mass index (BMI), hyperglycemia, and physical inactivity, substantially contribute to the burden of CRC across all levels of the SDI. Karavasiloglou et al. have shown that higher serum ionized calcium levels are negatively associated with the risk of CRC [30]. In addition to dietary factors, high BMI is a notable contributor to CRC mortality. Prior research has established the link between obesity and CRC [31], and a study by Li et al. confirmed that lifelong overweight/obesity increases the risk of CRC [32]. Furthermore, smoking has been confirmed as a definite risk factor for CRC. Studies have shown that smokers have a significantly higher risk of CRC than non-smokers. Harmful substances in tobacco can promote the progression of CRC by affecting DNA methylation, cell proliferation, and apoptosis in intestinal cells. The greater the amount of smoking and the longer the duration, the higher the risk [33,34]. Quitting smoking can reduce but not completely eliminate this risk.

Globally, higher CRC incidence and mortality rates in men than in women have been observed. This disparity may be attributable not only to differences in diet and lifestyle between sexes, such as smoking and alcohol consumption, but also to physiological differences [35]. Studies have indicated that sex hormone differences are one of the important causes of the gender disparity in CRC, with estrogen potentially having an inhibitory effect on the progression of CRC, while androgens may increase the risk of CRC [36]. Further research has found that gut metabolites in males exacerbate colorectal tumorigenesis through the glycerophospholipid metabolism pathway [37]. Given that men have a relatively higher risk of CRC, interventions for CRC in men, including screening, diagnosis, and treatment, should be prioritized.

The findings of this study are of significant importance to public health policy and clinical practice. The increasing incidence of CRC in younger age groups necessitates a re-evaluation of screening guidelines and public health campaigns. Current screening recommendations, which typically target individuals aged 50 years and older, may need to be adjusted to accommodate the changing demographics of CRC. For instance, Lansdorp-Vogelaar et al. have indicated that in high-risk populations such as those with a family history of CRC or those living in areas with high incidence rates, screening should commence at an earlier age [38]. Moreover, the substantial contribution of lifestyle factors to CRC risk highlights the need for comprehensive public health campaigns to promote healthy behaviors.

This study has several limitations. Firstly, variations in data-collection methods, reporting practices, and disease-coding standards across different regions may affect the precision of the study results. Secondly, the study did not take into account potential confounding factors that could influence observed trends in diverse populations, such as genetic susceptibility, family history of CRC, and specific dietary patterns. Thirdly, the analysis was based on aggregated data, lacking individual-level information, which restricted the capacity to conduct more detailed subgroup analyses and explore potential interactions between variables. Furthermore, with the study span from 2010 to 2021, the results may not fully capture the latest trends or the impact of emerging risk factors and interventions on the CRC burden. Finally, the regional classification based on the SDI might oversimplify the complex socio-economic and cultural factors affecting CRC epidemiology, necessitating further research to better understand regional differences and their underlying causes.

## Conclusion

The epidemiology of CRC globally presents a complex and evolving picture, characterized by rising incidence, falling mortality, and marked regional variations. Our study found that the burden of CRC is greater in men than in women, highlighting the need for early screening, diagnosis, and therapeutic interventions in men. Among various age groups, the incidence of CRC is highest in those aged over 70 years, although it is showing a declining trend, while the incidence in the 15–49 and 50–69 age groups is increasing, underscoring the need for targeted interventions. This study emphasizes the impact of lifestyle and socioeconomic factors on CRC trends and highlights the importance of promoting healthy behaviors and improving screening through public health measures. Addressing the increasing burden of CRC, particularly in younger populations and high-risk regions, requires a multifaceted approach involving policy, prevention, and medical advancements to mitigate its impact on global health.

## Supplementary Material

STable.docx

Figure S1.tif

## Data Availability

GBD study 2021 data resources were available online from the Global Health Data Exchange (GHDx) query tool (http://ghdx.healthdata.org/gbd-results-tool). All data generated or analyzed during this study are included in this article and supplementary material. Further details may be obtained from the corresponding author upon request.
