# Supplementary material for: Global, regional, and national trends in colorectal cancer from 2010 to 2021: an analysis of the global burden of disease study 2021
Source: Ann Med. 2025 Aug 1;57(1):2534098. doi: 10.1080/07853890.2025.2534098 (PMC12330823; doi:10.1080/07853890.2025.2534098)
Supplement: STable.docx [file IANN_A_2534098_SM1906.docx]

**Title: Global, regional, and national trends in colorectal cancer from 2010 to 2021: An analysis of the Global Burden of Disease Study 2021**

**Gerun Chen^1,2^, Jialin Wu^3,4^, Sibo Huang^5^,** **Zhenqi Gong^1,2^, Huaiming Wang^1*^**

Table of contents

[TableS1: 2](#_Toc191147138)

[TableS2: 5](#_Toc191147139)

[TableS3: 6](#_Toc191147140)

[TableS4: 13](#_Toc191147141)

TableS1: Global and regional numbers of incidence, mortality, and DALY for CRC in 2010 and 2021.

| **Location** | **2010 all-age number (95%UI)** | | | **2021 all-age number (95%UI)** | | |
| --- | --- | --- | --- | --- | --- | --- |
|  | **Incidence** | **Mortality** | **DALY** | **Incidence** | **Mortality** | **DALY** |
| Global | 2194143.250 (2001271.824, 2359390.088) | 820414.961 (767047.553, 851834.429) | 19687652.09 (18818729.49, 20373871.45) | 2194143.250 (2001271.824, 2359390.088) | 1044072.211 (950187.610, 1120169.335) | 24401100.18 (22689368.55, 26161517.73) |
| **SDI** |  |  |  |  |  |  |
| High SDI | 839754.506 (764451.138, 885156.390) | 293578.716 (265687.373, 308247.925) | 6158892.29 (5789543.88, 6411162.98) | 839754.506 (764451.138, 885156.390) | 336566.143 (299021.834, 359612.839) | 6705748.75 (6179255.70, 7070831.33) |
| High-middle SDI | 669659.584 (598305.510, 746397.458) | 252231.650 (238116.052, 263420.243) | 5971853.77 (5669573.50, 6253228.84) | 669659.584 (598305.510, 746397.458) | 308174.804 (278041.590, 338271.333) | 7079677.99 (6397925.01, 7847047.95) |
| Middle SDI | 526190.397 (462015.908, 595123.927) | 188015.839 (177140.756, 198781.514) | 5086648.56 (4793846.56, 5389861.33) | 526190.397 (462015.908, 595123.927) | 274871.222 (243369.107, 306694.039) | 7120502.07 (6308564.18, 7920507.32) |
| Low-middle SDI | 119416.292 (109344.124, 130918.557) | 63279.545 (58880.286, 68026.330) | 1814673.33 (1689676.90, 1945055.26) | 119416.292 (109344.124, 130918.557) | 91274.174 (83732.612, 99854.467) | 2564120.92 (2333600.66, 2815224.00) |
| Low SDI | 36649.311 (32707.360, 40883.463) | 22144.437 (20075.865, 24518.919) | 629220.66 (569638.17, 697302.26) | 36649.311 (32707.360, 40883.463) | 31847.591 (28503.900, 35514.274) | 901644.93 (799642.29, 1014005.07) |
| **Regions** |  |  |  |  |  |  |
| Andean Latin America | 8452.057 (6698.577, 10526.053) | 4161.540 (3654.902, 4717.080) | 98471.41 (86706.51, 111960.28) | 8452.057 (6698.577, 10526.053) | 5777.836 (4597.210, 7047.450) | 136183.21 (108534.69, 167981.61) |
| Australasia | 23280.743 (20501.569, 26412.689) | 6887.924 (6071.007, 7490.060) | 144630.55 (131774.48, 156868.47) | 23280.743 (20501.569, 26412.689) | 8275.772 (7178.893, 9408.206) | 166740.10 (147523.69, 186890.83) |
| Caribbean | 18481.225 (16074.151, 20935.797) | 6050.492 (5595.553, 6494.724) | 138967.50 (128502.07, 150620.92) | 18481.225 (16074.151, 20935.797) | 7881.536 (6866.692, 8970.236) | 179751.92 (155874.18, 205420.07) |
| Central Asia | 8892.968 (7941.559, 9809.245) | 5075.471 (4736.616, 5435.060) | 139356.19 (130683.79, 148481.37) | 8892.968 (7941.559, 9809.245) | 6146.247 (5492.549, 6771.900) | 169952.25 (151537.43, 187708.00) |
| Central Europe | 85866.578 (79290.327, 92786.951) | 46592.072 (44303.315, 48493.511) | 1030201.93 (990274.91, 1071112.49) | 85866.578 (79290.327, 92786.951) | 51843.486 (47752.159, 55681.287) | 1087562.43 (1003626.42, 1168378.10) |
| Central Latin America | 44551.535 (39665.469, 49720.643) | 13846.818 (13099.584, 14409.655) | 362639.58 (347766.15, 375048.54) | 44551.535 (39665.469, 49720.643) | 22934.275 (20341.655, 25521.139) | 594116.79 (529274.15, 662367.07) |
| Central Sub-Saharan Africa | 4206.451 (3206.030, 5573.585) | 2374.250 (1868.123, 2909.324) | 69884.41 (55354.42, 86318.52) | 4206.451 (3206.030, 5573.585) | 3686.773 (2802.235, 4932.004) | 109622.05 (82688.06, 146971.08) |
| East Asia | 684927.152 (559522.761, 823301.329) | 202958.933 (184824.546, 222625.050) | 5333451.31 (4833265.69, 5901101.21) | 684927.152 (559522.761, 823301.329) | 287880.009 (235559.365, 343280.486) | 7148995.32 (5822935.26, 8561078.94) |
| Eastern Europe | 113252.178 (104414.531, 122488.524) | 59892.900 (57548.765, 61723.034) | 1415963.63 (1368896.31, 1457513.04) | 113252.178 (104414.531, 122488.524) | 64373.247 (59115.498, 69822.450) | 1465104.15 (1343980.89, 1600585.46) |
| Eastern Sub-Saharan Africa | 17951.902 (15710.425, 20779.075) | 10798.127 (9935.882, 11805.101) | 303805.12 (279368.21, 331696.63) | 17951.902 (15710.425, 20779.075) | 15967.479 (13939.888, 18320.333) | 444252.95 (385799.78, 525458.32) |
| High-income Asia Pacific | 207276.974 (179498.139, 223331.926) | 62733.321 (54773.617, 66953.562) | 1272056.34 (1162547.33, 1341018.88) | 207276.974 (179498.139, 223331.926) | 80690.589 (67266.650, 88001.374) | 1441630.00 (1272495.98, 1549758.69) |
| High-income North America | 244681.446 (226550.009, 256376.909) | 78119.619 (70754.771, 82170.298) | 1743476.98 (1632838.87, 1821817.76) | 244681.446 (226550.009, 256376.909) | 85865.380 (77871.228, 90928.137) | 1907928.37 (1788110.92, 2003823.63) |
| North Africa and Middle East | 66086.551 (58129.632, 74936.328) | 25759.629 (23782.062, 27936.782) | 709251.27 (656897.88, 770140.33) | 66086.551 (58129.632, 74936.328) | 37394.507 (32758.786, 42274.127) | 1012651.60 (886198.88, 1154502.81) |
| Oceania | 481.600 (409.984, 560.140) | 263.054 (230.178, 298.112) | 8092.11 (6944.98, 9349.46) | 481.600 (409.984, 560.140) | 381.662 (324.226, 445.960) | 11642.97 (9764.00, 13750.35) |
| South Asia | 85115.134 (76613.731, 95247.802) | 44318.761 (40958.136, 47372.676) | 1304929.13 (1209285.60, 1400110.86) | 85115.134 (76613.731, 95247.802) | 66942.714 (60196.675, 74844.400) | 1908668.14 (1711982.01, 2155055.24) |
| Southeast Asia | 116941.670 (101259.791, 132256.456) | 53366.191 (47687.724, 58630.174) | 1504526.68 (1332335.62, 1668145.63) | 116941.670 (101259.791, 132256.456) | 79419.596 (68449.689, 89290.459) | 2166650.37 (1868880.14, 2456349.67) |
| Southern Latin America | 24746.824 (21921.766, 27561.451) | 14858.544 (13536.930, 16185.524) | 320889.56 (294308.78, 351470.35) | 24746.824 (21921.766, 27561.451) | 16117.019 (14307.191, 18001.796) | 349581.13 (311369.42, 391893.39) |
| Southern Sub-Saharan Africa | 7623.406 (6875.232, 8472.177) | 5014.339 (4722.235, 5304.904) | 136204.72 (128046.08, 144497.24) | 7623.406 (6875.232, 8472.177) | 6129.623 (5550.193, 6785.914) | 166061.78 (149959.40, 186753.43) |
| Tropical Latin America | 44244.626 (40859.427, 47143.628) | 20049.779 (18664.085, 21103.990) | 518110.48 (492633.59, 541368.73) | 44244.626 (40859.427, 47143.628) | 29414.564 (26980.718, 31338.903) | 744957.01 (694956.43, 786849.09) |
| Western Europe | 375461.835 (337713.278, 401850.236) | 149968.338 (133687.136, 158492.968) | 2938791.29 (2715565.07, 3087658.88) | 375461.835 (337713.278, 401850.236) | 156637.467 (136856.634, 169938.671) | 2912355.20 (2641021.36, 3112518.77) |
| Western Sub-Saharan Africa | 11620.394 (9673.144, 13673.399) | 7324.861 (6397.295, 8191.643) | 193951.91 (165997.71, 219670.31) | 11620.394 (9673.144, 13673.399) | 10312.429 (8668.115, 12099.874) | 276692.44 (224567.81, 328426.26) |

**DALY: Disability-adjusted life-year, CRC:** **colorectal cancer, UI: Uncertainty interval, SDI: Sociodemographic index**

TableS2: Age-stratified numbers and percentage change in incidence, mortality, and DALY for CRC between 2010 and 2021.

| **Age** | **Incidence (95% UI)** | | | **Mortality (95% UI)** | | | **DALY (95% UI)** | | |
| --- | --- | --- | --- | --- | --- | --- | --- | --- | --- |
|  | **2010** | **2021** | **PC, %,**  **(2010-2021)** | **2010** | **2021** | **PC, %,**  **(2010-2021)** | **2010** | **2021** | **PC, %,**  **(2010-2021)** |
| 15-49 years | 4.94  (4.75, 5.14) | 5.37  (4.91, 5.86) | 8.58  (-0.88, 19.28) | 2.06  (1.97, 2.15) | 2.01  (1.84, 2.19) | -2.03  (-9.78, 7.07) | 103.31  (99.01, 107.88) | 101.37  (92.85 , 110.18) | -1.88  (-9.71, 7.20) |
| 50-69 years | 63.33  (61.18, 65.29) | 65.47  (60.46, 70.97) | 3.39  (-4.45, 11.94) | 27.16  (26.18, 28.00) | 25.97  (24.13, 28.11) | -4.38  (-11.20, 3.42) | 845.84  (814.46, 873.93) | 803.91  (746.52, 868.22) | -4.96  (-11.84, 2.88) |
| 70+ years | 212.36  (191.83, 222.46) | 210.72  (187.94, 227.06) | -0.77  (-6.01, 4.36) | 127.40  (114.51, 133.83) | 119.64  (106.19, 128.90) | -6.09  (-10.82, -1.34) | 1933.46  (1770.25, 2023.43) | 1790.14  (1602.69, 1924.17) | -7.41  (-12.35, -2.43) |

**DALY: Disability-adjusted life-year, CRC: colorectal cancer, UI: Uncertainty interval, PC: Percentage change**

TableS3: ASIR、ASMR and ASDR of Colorectal Cancer’s disease by countries in 2021.

| **Location** | **ASIR (95% UI)** | **ASMR (95% UI)** | **ASDR (95% UI)** |
| --- | --- | --- | --- |
| Global | 25.61 (23.32, 27.52) | 12.40 (11.24,13.31) | 283.24 (263.11,303.33) |
| Afghanistan | 14.16 (6.79,21.48) | 12.30 (6.20,18.55) | 324.71 (146.82,503.12) |
| Albania | 11.48 (8.85,15.39) | 7.52 (5.79,9.81) | 156.46 (120.27,209.47) |
| Algeria | 6.95 (5.39,8.79) | 4.80 (3.78,5.99) | 99.94 (78.40,126.74) |
| American Samoa | 19.36 (16.14,23.75) | 15.48 (12.96,18.89) | 366.75 (306.83,450.38) |
| Andorra | 44.68 (30.29,60.44) | 15.91 (11.45,21.20) | 344.77 (238.41,466.30) |
| Angola | 9.04 (6.63,11.83) | 8.53 (6.26,11.21) | 203.35 (148.46,271.46) |
| Antigua and Barbuda | 32.44 (29.54,35.42) | 14.95 (13.61,16.34) | 320.56 (294.20,346.47) |
| Argentina | 28.79 (25.21,32.36) | 19.44 (17.08,21.88) | 443.27 (387.89,500.57) |
| Armenia | 19.55 (17.07,22.33) | 13.12 (11.50,15.07) | 301.97 (265.01,345.93) |
| Australia | 42.68 (37.58,48.59) | 13.98 (12.19,15.90) | 315.46 (278.06,356.65) |
| Austria | 29.27 (25.66,33.38) | 11.80 (10.26,13.52) | 253.07 (223.60,287.33) |
| Azerbaijan | 9.79 (7.42,12.32) | 7.18 (5.46,8.98) | 184.50 (138.24,234.39) |
| Bahamas | 36.11 (28.86,44.63) | 18.47 (15.15,22.54) | 444.71 (357.74,553.53) |
| Bahrain | 18.79 (15.12,25.02) | 10.98 (8.94,14.46) | 227.10 (183.24,307.76) |
| Bangladesh | 4.41 (3.29,6.03) | 3.58 (2.71,4.89) | 88.88 (65.03,124.89) |
| Barbados | 50.65 (39.74,62.59) | 22.93 (18.10,27.93) | 496.33 (387.48,616.82) |
| Belarus | 36.09 (27.38,45.99) | 17.32 (13.20,21.73) | 415.90 (314.40,530.09) |
| Belgium | 36.35 (31.83,40.73) | 14.13 (12.20,15.98) | 301.55 (264.48,338.67) |
| Belize | 16.66 (14.53,19.41) | 9.20 (7.95,10.64) | 218.78 (188.42,255.02) |
| Benin | 5.85 (4.47,7.48) | 5.66 (4.39,7.17) | 123.35 (93.20,162.85) |
| Bermuda | 61.79 (51.46,77.11) | 18.37 (15.27,22.59) | 396.54 (329.53,490.21) |
| Bhutan | 5.26 (3.97,6.84) | 4.41 (3.31,5.68) | 106.36 (78.43,138.93) |
| Bolivia(Plurinational State of) | 16.31 (11.17,22.63) | 14.04 (9.59,19.38) | 308.93 (211.81,431.97) |
| Bosnia and Herzegovina | 30.79 (23.78,38.32) | 18.90 (14.84,23.39) | 423.43 (323.22,528.91) |
| Botswana | 11.05 (8.43,14.95) | 10.08 (7.76,13.38) | 226.73 (167.80,313.81) |
| Brazil | 17.23 (15.86,18.39) | 11.60 (10.60,12.37) | 287.32 (267.76,303.88) |
| Brunei Darussalam | 30.04 (25.37,35.53) | 20.16 (17.04,23.91) | 466.43 (393.67,547.69) |
| Bulgaria | 46.04 (37.65,55.79) | 25.71 (20.96,30.73) | 605.00 (493.22,726.67) |
| Burkina Faso | 6.26 (4.71,7.98) | 6.17 (4.70,7.85) | 134.58 (99.55,173.68) |
| Burundi | 8.43 (6.15,11.61) | 8.33 (6.11,11.45) | 193.62 (138.14,275.06) |
| Cabo Verde | 10.83 (8.47,13.09) | 9.12 (7.01,10.81) | 179.56 (140.03,216.00) |
| Cambodia | 16.99 (12.67,21.96) | 14.37 (10.80,18.37) | 353.09 (262.55,456.87) |
| Cameroon | 7.91 (5.68,10.58) | 7.48 (5.44,9.94) | 167.20 (116.22,226.65) |
| Canada | 43.29 (38.58,47.88) | 14.15 (12.47,15.80) | 317.47 (285.08,351.22) |
| Central African Republic | 9.11 (6.39,12.71) | 9.12 (6.40,12.86) | 229.71 (154.29,327.17) |
| Chad | 6.32 (4.73,8.20) | 6.29 (4.79,8.11) | 142.86 (106.23,186.72) |
| Chile | 24.09 (21.07,27.25) | 13.13 (11.54,14.80) | 292.24 (258.58,329.15) |
| China | 31.44 (25.53,37.97) | 13.64 (11.09,16.31) | 331.73 (267.78,400.70) |
| Colombia | 22.28 (18.37,27.26) | 10.49 (8.66,12.68) | 258.01 (212.77,310.81) |
| Comoros | 10.56 (7.55,13.96) | 10.04 (7.17,13.40) | 230.84 (164.12,314.31) |
| Congo | 10.97 (8.68,13.79) | 10.10 (8.03,12.53) | 242.56 (187.26,310.42) |
| Cook Islands | 9.43 (7.53,11.93) | 5.68 (4.59,7.09) | 129.05 (102.56,164.07) |
| Costa Rica | 35.99 (31.18,42.31) | 15.13 (13.09,17.48) | 370.71 (321.50,430.24) |
| Côte d'Ivoire | 4.31 (3.27,5.89) | 4.00 (3.06,5.39) | 92.14 (67.87,128.91) |
| Croatia | 49.75 (42.40,57.25) | 24.29 (20.78,27.97) | 524.86 (450.88,601.57) |
| Cuba | 48.47 (40.77,56.59) | 17.47 (14.89,20.22) | 388.50 (327.70,454.12) |
| Cyprus | 34.23 (28.58,40.59) | 12.75 (10.76,14.93) | 247.79 (208.91,294.36) |
| Czechia | 40.24 (33.58,47.83) | 20.16 (16.98,23.84) | 446.39 (371.66,531.64) |
| Democratic People's Republic of Korea | 15.43 (10.42,22.53) | 10.51 (7.05,15.44) | 283.00 (189.17,427.86) |
| Democratic Republic of the Congo | 6.90 (4.78,9.92) | 6.59 (4.52,9.67) | 156.42 (107.84,227.19) |
| Denmark | 43.45 (37.30,49.33) | 18.97 (16.41,21.34) | 384.64 (333.80,432.46) |
| Djibouti | 13.50 (9.66,18.59) | 12.66 (9.13,17.12) | 290.23 (205.38,408.80) |
| Dominica | 24.62 (20.79,29.66) | 15.18 (12.81,17.99) | 331.61 (278.95,402.29) |
| Dominican Republic | 15.45 (12.13,19.74) | 9.14 (7.10,11.73) | 215.86 (168.90,277.61) |
| Ecuador | 13.04 (10.17,16.50) | 9.46 (7.42,11.88) | 215.97 (166.95,275.35) |
| Egypt | 12.57 (10.41,15.33) | 8.78 (7.29,10.58) | 212.77 (174.34,258.80) |
| El Salvador | 14.81 (12.01,18.31) | 8.11 (6.54,9.90) | 206.97 (165.35,253.14) |
| Equatorial Guinea | 12.25 (8.33,17.07) | 10.49 (7.24,14.52) | 245.07 (165.22,345.96) |
| Eritrea | 12.11 (9.05,15.78) | 11.82 (8.89,15.28) | 282.31 (208.73,376.44) |
| Estonia | 40.93 (33.39,48.65) | 17.80 (14.52,20.87) | 382.11 (310.79,447.00) |
| Eswatini | 16.74 (10.96,22.46) | 15.24 (10.05,20.14) | 378.16 (242.58,517.06) |
| Ethiopia | 16.41 (13.70,19.54) | 15.91 (13.33,19.01) | 343.04 (284.97,415.16) |
| Fiji | 11.22 (8.35,14.50) | 9.72 (7.29,12.58) | 226.96 (167.30,295.51) |
| Finland | 30.63 (26.84,34.28) | 11.32 (9.78,12.77) | 243.95 (215.21,273.85) |
| France | 41.80 (36.04,47.25) | 15.12 (12.88,17.10) | 324.49 (282.40,367.06) |
| Gabon | 15.45 (11.31,20.01) | 13.54 (9.99,17.31) | 319.35 (230.62,421.91) |
| Gambia | 3.31 (2.54,4.27) | 3.05 (2.34,3.93) | 70.75 (53.17,92.31) |
| Georgia | 18.73 (16.08,21.69) | 13.30 (11.42,15.29) | 328.78 (281.26,381.66) |
| Germany | 38.53 (33.72,43.31) | 14.63 (12.49,16.47) | 323.01 (286.33,360.79) |
| Ghana | 7.41 (5.81,9.21) | 6.89 (5.39,8.51) | 150.82 (117.21,189.48) |
| Greece | 33.38 (29.96,37.16) | 14.03 (12.29,15.52) | 297.27 (268.42,326.68) |
| Greenland | 38.22 (32.36,45.88) | 24.60 (20.85,29.54) | 572.67 (484.22,684.00) |
| Grenada | 31.36 (26.48,36.76) | 17.32 (14.67,20.21) | 387.52 (324.20,453.25) |
| Guam | 16.97 (14.62,19.62) | 10.23 (8.77,11.85) | 291.86 (253.74,332.83) |
| Guatemala | 9.79 (8.42,11.48) | 6.93 (5.98,8.05) | 168.37 (143.72,196.84) |
| Guinea | 4.85 (3.63,6.48) | 4.69 (3.52,6.25) | 110.34 (80.27,151.15) |
| Guinea-Bissau | 8.48 (6.36,10.64) | 8.39 (6.31,10.47) | 195.76 (146.32,248.83) |
| Guyana | 19.48 (14.82,25.11) | 13.25 (10.17,16.90) | 325.81 (246.26,422.11) |
| Haiti | 16.53 (11.29,23.36) | 13.83 (9.56,19.25) | 325.88 (222.11,464.01) |
| Honduras | 8.32 (6.62,10.76) | 6.27 (4.99,8.15) | 147.97 (116.10,194.46) |
| Hungary | 49.51 (40.97,59.24) | 26.01 (21.73,31.13) | 614.96 (519.37,736.20) |
| Iceland | 32.67 (28.14,38.09) | 11.19 (9.38,13.03) | 239.60 (207.01,275.80) |
| India | 5.69 (5.05,6.45) | 4.60 (4.09,5.21) | 120.93 (107.62,137.15) |
| Indonesia | 15.03 (11.40,18.74) | 12.39 (9.50,15.30) | 297.58 (223.31,371.79) |
| Iran (Islamic Republic of) | 13.19 (11.44,14.76) | 7.37 (6.31,8.19) | 174.30 (149.14,193.93) |
| Iraq | 10.04 (7.60,12.68) | 6.33 (4.84,7.83) | 154.27 (116.39,193.92) |
| Ireland | 40.89 (35.16,46.57) | 14.38 (12.21,16.40) | 308.17 (267.90,349.96) |
| Israel | 29.81 (25.05,34.37) | 13.35 (10.94,15.48) | 270.88 (231.00,310.26) |
| Italy | 39.65 (35.64,42.66) | 14.28 (12.57,15.43) | 309.66 (284.37,331.08) |
| Jamaica | 31.70 (23.99,42.39) | 15.09 (11.62,19.81) | 353.13 (266.97,471.23) |
| Japan | 48.70 (44.07,51.75) | 15.89 (13.97,16.97) | 358.44 (327.93,378.99) |
| Jordan | 15.51 (11.84,20.96) | 8.78 (6.70,11.64) | 205.95 (158.78,277.99) |
| Kazakhstan | 15.43 (13.06,17.98) | 10.53 (8.90,12.23) | 261.66 (224.14,303.16) |
| Kenya | 7.89 (6.48,9.79) | 7.18 (5.91,8.86) | 171.46 (141.02,215.33) |
| Kiribati | 9.43 (7.43,12.48) | 9.00 (7.25,11.87) | 223.53 (170.29,299.18) |
| Kuwait | 19.05 (15.40,23.23) | 8.67 (7.06,10.72) | 194.76 (155.30,241.81) |
| Kyrgyzstan | 9.61 (7.84,11.52) | 7.03 (5.66,8.56) | 181.61 (148.95,218.19) |
| Lao People's Democratic Republic | 14.70 (10.62,19.11) | 13.03 (9.55,16.89) | 323.48 (229.50,429.29) |
| Latvia | 30.02 (24.50,35.79) | 16.66 (13.60,19.90) | 380.02 (310.97,453.77) |
| Lebanon | 20.30 (16.48,25.89) | 10.99 (8.98,14.02) | 236.40 (193.55,298.75) |
| Lesotho | 14.06 (10.03,19.74) | 13.47 (9.64,19.03) | 328.58 (228.45,473.52) |
| Liberia | 5.94 (3.78,8.87) | 5.58 (3.60,8.29) | 124.19 (77.79,186.56) |
| Libya | 21.49 (15.65,28.68) | 13.95 (10.27,18.59) | 332.66 (242.52,449.39) |
| Lithuania | 30.85 (25.55,36.25) | 16.92 (13.90,19.95) | 380.34 (315.77,449.63) |
| Luxembourg | 37.51 (33.20,42.15) | 14.62 (12.82,16.25) | 299.97 (264.08,334.57) |
| Madagascar | 8.48 (6.16,10.94) | 8.12 (5.92,10.46) | 193.53 (139.73,252.26) |
| Malawi | 4.84 (3.81,6.50) | 4.55 (3.57,6.08) | 109.53 (85.08,150.62) |
| Malaysia | 26.12 (22.65,29.53) | 18.01 (15.58,20.27) | 418.71 (364.59,469.07) |
| Maldives | 6.59 (5.32,8.14) | 4.37 (3.53,5.34) | 94.22 (76.20,117.52) |
| Mali | 7.43 (5.79,9.30) | 7.10 (5.55,8.96) | 164.24 (125.81,208.77) |
| Malta | 34.01 (28.75,38.87) | 13.62 (11.31,15.67) | 292.12 (249.33,335.41) |
| Marshall Islands | 13.31 (9.85,17.40) | 12.01 (9.12,15.47) | 299.61 (218.73,393.72) |
| Mauritania | 7.93 (5.79,10.59) | 7.09 (5.18,9.50) | 152.11 (111.06,206.82) |
| Mauritius | 21.62 (19.58,23.31) | 14.25 (12.99,15.31) | 354.37 (324.38,380.39) |
| Mexico | 16.16 (14.20,18.20) | 8.78 (7.77,9.81) | 223.11 (196.93,250.75) |
| Micronesia (Federated States of) | 14.14 (10.28,19.12) | 12.42 (9.00,16.65) | 303.82 (219.46,415.67) |
| Monaco | 68.33 (54.05,83.19) | 25.23 (20.13,30.60) | 551.88 (440.65,676.50) |
| Mongolia | 10.01 (7.82,12.45) | 8.12 (6.35,10.04) | 207.23 (162.14,256.08) |
| Montenegro | 31.46 (26.15,38.30) | 17.74 (14.86,20.97) | 378.80 (314.03,462.92) |
| Morocco | 11.56 (8.38,15.19) | 8.33 (6.20,10.64) | 200.91 (146.47,263.30) |
| Mozambique | 4.08 (3.11,5.07) | 4.15 (3.15,5.18) | 86.42 (64.64,109.87) |
| Myanmar | 13.71 (10.09,17.58) | 11.56 (8.58,14.83) | 286.48 (209.56,372.14) |
| Namibia | 7.40 (5.50,9.48) | 6.37 (4.83,8.03) | 157.04 (113.73,204.68) |
| Nauru | 19.80 (12.26,26.38) | 16.98 (10.74,22.30) | 428.75 (258.39,574.72) |
| Nepal | 4.85 (3.61,6.39) | 4.14 (3.09,5.45) | 103.55 (76.75,136.66) |
| Netherlands | 69.80 (62.21,76.79) | 19.99 (17.63,22.18) | 444.07 (399.16,488.14) |
| New Zealand | 51.08 (44.46,58.12) | 18.15 (15.68,20.59) | 390.26 (342.89,440.26) |
| Nicaragua | 10.08 (8.17,12.28) | 5.89 (4.81,7.11) | 145.04 (116.78,176.92) |
| Niger | 5.06 (3.63,6.78) | 5.05 (3.66,6.74) | 110.28 (78.25,149.79) |
| Nigeria | 6.08 (4.88,7.47) | 5.78 (4.76,6.96) | 126.42 (98.66,158.92) |
| Niue | 15.64 (12.56,18.92) | 12.03 (9.73,14.51) | 281.49 (224.77,345.69) |
| North Macedonia | 29.07 (23.07,35.39) | 19.26 (15.65,23.16) | 404.98 (321.47,494.20) |
| Northern Mariana Islands | 22.37 (19.09,25.01) | 15.57 (13.29,17.46) | 358.92 (305.62,398.44) |
| Norway | 47.11 (42.42,50.84) | 18.12 (15.88,19.54) | 369.37 (337.20,395.53) |
| Oman | 6.43 (4.96,8.11) | 3.65 (2.85,4.55) | 81.01 (62.04,103.75) |
| Pakistan | 6.88 (5.67,8.56) | 6.29 (5.22,7.80) | 154.08 (125.46,192.31) |
| Palau | 16.94 (13.16,21.11) | 13.55 (10.47,16.95) | 290.20 (223.31,362.20) |
| Palestine | 23.13 (19.17,27.26) | 15.16 (12.68,17.71) | 342.54 (282.99,404.33) |
| Panama | 27.81 (21.29,34.22) | 11.12 (8.57,13.52) | 272.38 (209.78,334.02) |
| Papua New Guinea | 3.94 (3.08,4.91) | 3.56 (2.80,4.41) | 91.65 (70.55,115.21) |
| Paraguay | 14.76 (11.38,18.97) | 10.57 (8.18,13.31) | 243.96 (188.24,310.59) |
| Peru | 14.65 (10.91,19.31) | 9.32 (6.95,12.00) | 210.60 (157.02,278.12) |
| Philippines | 16.55 (14.01,19.38) | 13.18 (11.18,15.35) | 342.33 (287.17,402.30) |
| Poland | 35.32 (31.88,38.43) | 24.35 (21.86,26.45) | 522.54 (474.13,567.42) |
| Portugal | 42.44 (36.62,48.89) | 17.32 (14.85,19.68) | 383.54 (331.36,439.81) |
| Puerto Rico | 46.52 (38.42,56.28) | 13.48 (11.26,16.21) | 337.60 (281.28,406.32) |
| Qatar | 23.29 (17.72,31.38) | 11.38 (8.70,15.34) | 232.78 (176.63,318.28) |
| Republic of Korea | 35.48 (29.08,41.81) | 12.56 (10.32,14.83) | 267.43 (223.39,313.79) |
| Republic of Moldova | 31.64 (26.71,37.44) | 18.07 (15.29,21.24) | 450.95 (381.09,528.67) |
| Romania | 38.46 (32.20,45.92) | 21.09 (17.92,24.89) | 504.85 (430.58,593.48) |
| Russian Federation | 34.18 (31.30,37.02) | 18.99 (17.47,20.56) | 436.30 (400.45,472.48) |
| Rwanda | 10.01 (7.04,13.31) | 9.49 (6.69,12.68) | 216.77 (151.91,293.35) |
| Saint Kitts and Nevis | 31.64 (26.54,37.16) | 17.13 (14.45,19.91) | 370.59 (309.14,438.31) |
| Saint Lucia | 20.58 (16.82,24.97) | 11.03 (9.08,13.30) | 244.76 (199.83,298.55) |
| Saint Vincent and the Grenadines | 22.65 (19.93,26.01) | 13.23 (11.65,15.05) | 301.78 (264.09,348.32) |
| Samoa | 11.59 (9.31,14.21) | 9.35 (7.59,11.42) | 221.44 (176.93,274.29) |
| San Marino | 30.45 (20.08,43.72) | 11.13 (7.46,15.39) | 239.27 (152.40,336.44) |
| Sao Tome and Principe | 12.15 (9.98,14.94) | 10.85 (8.98,13.19) | 231.19 (188.42,286.26) |
| Saudi Arabia | 14.88 (12.12,18.16) | 7.64 (6.29,9.36) | 190.23 (152.15,235.94) |
| Senegal | 7.26 (5.59,9.41) | 6.90 (5.27,8.92) | 150.77 (114.40,198.68) |
| Serbia | 35.31 (28.47,43.10) | 21.32 (17.31,25.92) | 484.60 (388.90,590.27) |
| Seychelles | 27.82 (23.85,31.88) | 19.96 (17.19,22.88) | 469.22 (403.01,539.66) |
| Sierra Leone | 5.56 (4.16,7.37) | 5.36 (4.03,7.11) | 119.43 (87.67,161.53) |
| Singapore | 34.92 (30.08,40.08) | 12.43 (10.73,14.16) | 272.39 (239.77,309.64) |
| Slovakia | 53.35 (43.82,63.47) | 24.71 (20.25,29.54) | 563.00 (463.95,671.00) |
| Slovenia | 34.16 (27.82,42.22) | 16.18 (13.05,19.72) | 337.40 (273.75,416.54) |
| Solomon Islands | 10.63 (7.70,14.12) | 9.73 (7.09,12.79) | 243.85 (172.74,326.51) |
| Somalia | 9.93 (6.34,14.70) | 9.92 (6.38,14.54) | 243.25 (156.39,363.26) |
| South Africa | 13.46 (12.07,14.96) | 11.19 (10.02,12.42) | 262.56 (235.23,295.79) |
| South Sudan | 12.06 (8.35,15.90) | 11.53 (8.11,15.14) | 273.52 (191.51,363.38) |
| Spain | 46.22 (40.40,51.48) | 16.59 (14.25,18.69) | 361.91 (315.71,403.81) |
| Sri Lanka | 7.54 (4.99,10.49) | 4.52 (3.03,6.20) | 109.54 (71.59,153.93) |
| Sudan | 9.04 (6.06,12.80) | 6.95 (4.73,9.80) | 174.14 (115.92,252.47) |
| Suriname | 22.20 (16.99,28.06) | 13.84 (10.52,17.44) | 333.10 (255.41,418.25) |
| Sweden | 34.34 (29.04,40.05) | 14.37 (11.88,16.75) | 293.06 (249.23,339.71) |
| Switzerland | 28.11 (24.03,32.46) | 10.36 (8.65,12.00) | 219.17 (188.16,250.76) |
| Syrian Arab Republic | 10.56 (7.75,14.15) | 6.41 (4.73,8.55) | 150.89 (109.66,207.89) |
| Taiwan (Province of China) | 51.62 (45.24,57.72) | 21.86 (18.95,24.57) | 503.50 (445.35,558.95) |
| Tajikistan | 5.56 (4.29,6.93) | 4.69 (3.63,5.88) | 122.52 (92.92,159.66) |
| Thailand | 26.25 (19.95,33.54) | 14.89 (11.43,18.95) | 380.26 (287.87,486.34) |
| Timor-Leste | 10.66 (8.11,13.78) | 9.40 (7.23,12.26) | 227.22 (171.70,296.93) |
| Togo | 6.61 (4.53,9.15) | 6.26 (4.33,8.62) | 139.97 (92.93,195.14) |
| Tokelau | 12.79 (9.97,16.58) | 10.03 (7.75,12.86) | 240.72 (187.63,314.76) |
| Tonga | 8.93 (6.93,11.36) | 7.22 (5.65,9.12) | 163.21 (125.84,209.31) |
| Trinidad and Tobago | 30.36 (23.02,38.79) | 14.56 (11.18,18.53) | 355.66 (268.26,457.15) |
| Tunisia | 13.13 (9.49,17.88) | 7.52 (5.47,10.00) | 173.62 (124.24,238.55) |
| Turkey | 22.86 (18.36,28.06) | 12.93 (10.42,15.75) | 299.82 (240.50,368.43) |
| Turkmenistan | 6.79 (5.11,8.87) | 5.18 (3.97,6.74) | 140.91 (106.84,184.79) |
| Tuvalu | 12.64 (10.21,15.89) | 10.88 (8.76,13.79) | 263.31 (209.89,336.45) |
| Uganda | 11.44 (8.87,14.84) | 10.64 (8.29,13.83) | 252.54 (192.21,337.66) |
| Ukraine | 25.12 (18.71,32.57) | 15.67 (11.64,20.17) | 397.57 (288.28,518.22) |
| United Arab Emirates | 18.79 (14.27,26.88) | 13.05 (9.99,18.53) | 264.33 (200.81,376.61) |
| United Kingdom | 38.75 (35.98,40.37) | 15.72 (14.30,16.51) | 335.26 (314.03,348.83) |
| United Republic of Tanzania | 10.15 (7.58,12.91) | 9.50 (7.18,12.14) | 220.30 (162.36,289.53) |
| United States of America | 38.17 (35.56,39.98) | 12.79 (11.72,13.51) | 315.59 (298.11,330.02) |
| United States Virgin Islands | 30.36 (23.40,38.83) | 13.95 (10.72,17.67) | 330.77 (254.83,422.05) |
| Uruguay | 43.22 (38.45,48.54) | 27.46 (24.25,30.91) | 598.78 (533.29,672.27) |
| Uzbekistan | 6.34 (5.11,7.83) | 4.82 (3.86,5.91) | 131.07 (105.91,160.54) |
| Vanuatu | 11.18 (8.62,14.41) | 10.23 (7.90,13.11) | 252.45 (192.41,326.02) |
| Venezuela (Bolivarian Republic of) | 18.16 (13.38,23.62) | 10.25 (7.64,13.20) | 254.94 (187.83,332.69) |
| Viet Nam | 19.08 (14.66,23.00) | 12.12 (9.45,14.63) | 299.88 (228.83,365.46) |
| Yemen | 8.33 (5.43,11.95) | 6.91 (4.55,9.83) | 169.25 (111.11,246.93) |
| Zambia | 14.58 (9.44,26.19) | 13.54 (8.97,23.41) | 329.22 (207.45,621.59) |
| Zimbabwe | 14.51 (11.28,18.18) | 13.76 (10.88,17.01) | 333.74 (255.00,427.34) |

**ASIR:** **Age-standardized incidence rates, ASDR: Age-standardized mortality rates, ASDR: Age-standardized DALY rates, DALY: Disability-adjusted life-year, UI: Uncertainty interval**

TableS4: Global Trends of EAPCs for CRC Incidence, Mortality, and DALY from 2010 to 2021

| **Location** | **EAPCs (95% UI)** | | |
| --- | --- | --- | --- |
|  | **Incidence** | **Mortality** | **DALYs** |
| Afghanistan | 0.78 (0.74, 0.82) | 0.30 (0.26, 0.35) | 0.21 (0.15, 0.26) |
| Albania | 1.52 (0.88, 2.16) | 1.14 (0.53, 1.74) | 0.77 (0.07, 1.47) |
| Algeria | 0.81 (0.71, 0.90) | -0.29 (-0.36, -0.21) | -0.29 (-0.37, -0.22) |
| American Samoa | 0.78 (0.42, 1.14) | 0.56 (0.20, 0.92) | 0.55 (0.19, 0.91) |
| Andorra | -1.62 (-2.69, -0.54) | -1.93 (-2.91, -0.94) | -2.00 (-3.03, -0.95) |
| Angola | 1.02 (0.84, 1.20) | 0.64 (0.47, 0.82) | 0.47 (0.30, 0.65) |
| Antigua and Barbuda | 0.46 (-0.04, 0.96) | -0.33 (-0.90, 0.25) | -0.12 (-0.70, 0.46) |
| Argentina | -0.66 (-1.18, -0.15) | -1.32 (-1.86, -0.78) | -1.23 (-1.74, -0.72) |
| Armenia | 0.79 (0.38, 1.20) | 0.21 (-0.23, 0.65) | -0.15 (-0.52, 0.22) |
| Australia | -1.12 (-1.49, -0.75) | -1.27 (-1.65, -0.88) | -1.21 (-1.60, -0.82) |
| Austria | -1.83 (-2.10, -1.56) | -2.41 (-2.61, -2.21) | -2.46 (-2.70, -2.22) |
| Azerbaijan | -0.61 (-1.09, -0.12) | -1.27 (-1.75, -0.79) | -1.44 (-1.82, -1.07) |
| Bahamas | 0.01 (-0.35, 0.38) | -0.26 (-0.73, 0.20) | -0.27 (-0.63, 0.09) |
| Bahrain | -0.46 (-1.20, 0.28) | -2.09 (-3.16, -1.01) | -1.57 (-2.29, -0.83) |
| Bangladesh | 0.39 (-0.35, 1.12) | -0.35 (-1.20, 0.50) | -0.52 (-1.11, 0.08) |
| Barbados | 0.16 (-0.21, 0.52) | -0.29 (-0.68, 0.09) | -0.33 (-0.70, 0.04) |
| Belarus | 2.03 (1.52, 2.54) | 0.27 (-0.30, 0.83) | 0.22 (-0.45, 0.89) |
| Belgium | -1.85 (-2.37, -1.34) | -2.52 (-2.88, -2.16) | -2.72 (-3.17, -2.27) |
| Belize | 0.35 (-0.09, 0.80) | -0.21 (-0.65, 0.24) | -0.18 (-0.65, 0.30) |
| Benin | 0.11 (-0.07, 0.30) | -0.09 (-0.28, 0.11) | -0.20 (-0.44, 0.03) |
| Bermuda | -0.52 (-0.82, -0.22) | -0.70 (-0.98, -0.41) | -0.84 (-1.17, -0.51) |
| Bhutan | 1.14 (1.04, 1.25) | 0.51 (0.45, 0.57) | 0.27 (0.21, 0.33) |
| Bolivia (Plurinational State of) | 0.70 (0.59, 0.80) | 0.16 (0.07, 0.25) | 0.12 (0.06, 0.18) |
| Bosnia and Herzegovina | 0.57 (0.19, 0.96) | 0.31 (-0.08, 0.70) | -0.03 (-0.44, 0.39) |
| Botswana | -1.41 (-1.83, -0.99) | -1.83 (-2.24, -1.41) | -2.09 (-2.51, -1.66) |
| Brazil | 1.07 (0.92, 1.22) | 0.28 (0.12, 0.43) | 0.38 (0.25, 0.51) |
| Brunei Darussalam | 0.24 (-0.70, 1.19) | -0.24 (-1.46, 0.99) | 0.15 (-0.69, 1.01) |
| Bulgaria | 1.79 (1.18, 2.40) | 1.09 (0.53, 1.66) | 1.04 (0.52, 1.57) |
| Burkina Faso | 0.54 (0.18, 0.91) | 0.49 (0.13, 0.86) | 0.53 (0.09, 0.98) |
| Burundi | 0.33 (0.26, 0.40) | 0.23 (0.16, 0.29) | 0.08 (0.02, 0.13) |
| Cabo Verde | 3.80 (3.06, 4.54) | 3.50 (2.67, 4.34) | 2.86 (2.11, 3.61) |
| Cambodia | 1.26 (1.18, 1.35) | 0.70 (0.63, 0.78) | 0.49 (0.45, 0.54) |
| Cameroon | 0.13 (0.02, 0.25) | -0.20 (-0.35, -0.04) | -0.39 (-0.57, -0.22) |
| Canada | -1.52 (-1.85, -1.20) | -1.78 (-2.06, -1.51) | -1.58 (-1.86, -1.30) |
| Central African Republic | -0.62 (-0.74, -0.50) | -0.67 (-0.80, -0.55) | -0.71 (-0.84, -0.58) |
| Chad | 0.55 (0.48, 0.62) | 0.39 (0.31, 0.47) | 0.34 (0.28, 0.40) |
| Chile | 1.13 (0.49, 1.76) | -0.21 (-0.81, 0.40) | 0.16 (-0.41, 0.74) |
| China | 1.95 (1.68, 2.22) | -0.20 (-0.50, 0.10) | 0.00 (-0.29, 0.30) |
| Colombia | 2.46 (1.83, 3.08) | 0.92 (0.38, 1.47) | 1.10 (0.53, 1.66) |
| Comoros | 0.93 (0.76, 1.11) | 0.77 (0.61, 0.94) | 0.90 (0.58, 1.23) |
| Congo | 0.54 (0.37, 0.72) | 0.16 (-0.01, 0.33) | 0.08 (-0.09, 0.25) |
| Cook Islands | 0.85 (0.68, 1.03) | 0.03 (-0.16, 0.22) | 0.07 (-0.14, 0.28) |
| Costa Rica | 2.75 (2.00, 3.50) | 1.51 (0.82, 2.22) | 1.98 (1.35, 2.62) |
| Croatia | 0.43 (-0.23, 1.09) | -0.47 (-1.11, 0.18) | -0.73 (-1.37, -0.08) |
| Cuba | 2.13 (1.73, 2.54) | 1.04 (0.71, 1.38) | 1.22 (0.85, 1.58) |
| Cyprus | -1.60 (-2.11, -1.09) | -2.14 (-2.55, -1.72) | -2.09 (-2.49, -1.68) |
| Czechia | -1.50 (-1.86, -1.14) | -1.87 (-2.24, -1.49) | -2.06 (-2.44, -1.68) |
| Côte d'Ivoire | 0.02 (-0.21, 0.25) | -0.39 (-0.65, -0.13) | -0.34 (-0.71, 0.03) |
| Democratic People's Republic of Korea | -0.31 (-0.37, -0.25) | -1.13 (-1.20, -1.07) | -0.85 (-0.88, -0.82) |
| Democratic Republic of the Congo | 1.13 (0.99, 1.27) | 0.80 (0.65, 0.95) | 0.75 (0.58, 0.92) |
| Denmark | -1.23 (-1.56, -0.90) | -1.59 (-1.86, -1.31) | -1.98 (-2.23, -1.73) |
| Djibouti | 0.82 (0.70, 0.93) | 0.51 (0.42, 0.59) | 0.20 (0.11, 0.30) |
| Dominica | 0.06 (-0.10, 0.22) | -0.05 (-0.25, 0.14) | 0.01 (-0.24, 0.27) |
| Dominican Republic | 0.80 (0.54, 1.06) | 0.11 (-0.21, 0.43) | 0.61 (0.09, 1.14) |
| Ecuador | 0.24 (-0.14, 0.63) | -0.79 (-1.15, -0.43) | -0.50 (-0.94, -0.05) |
| Egypt | 0.93 (0.56, 1.29) | -0.14 (-0.69, 0.42) | -0.07 (-0.57, 0.44) |
| El Salvador | 2.08 (1.34, 2.82) | 0.98 (0.27, 1.70) | 1.20 (0.40, 2.02) |
| Equatorial Guinea | 1.62 (1.32, 1.92) | 1.06 (0.78, 1.33) | 1.02 (0.70, 1.34) |
| Eritrea | 0.35 (0.29, 0.42) | 0.18 (0.14, 0.22) | -0.01 (-0.07, 0.05) |
| Estonia | 1.79 (1.20, 2.39) | 0.92 (0.33, 1.51) | 0.66 (0.13, 1.19) |
| Eswatini | -1.16 (-1.34, -0.99) | -1.56 (-1.80, -1.33) | -1.73 (-1.84, -1.62) |
| Ethiopia | 0.91 (0.65, 1.18) | 0.53 (0.30, 0.76) | 0.29 (-0.00, 0.58) |
| Fiji | 1.07 (0.87, 1.28) | 0.80 (0.54, 1.06) | 0.67 (0.45, 0.90) |
| Finland | 0.44 (-0.04, 0.93) | -0.17 (-0.63, 0.30) | -0.46 (-0.84, -0.08) |
| France | -0.69 (-1.03, -0.35) | -1.59 (-1.80, -1.38) | -1.56 (-1.80, -1.32) |
| Gabon | 0.84 (0.71, 0.96) | 0.27 (0.15, 0.39) | 0.16 (0.04, 0.29) |
| Gambia | 1.68 (1.15, 2.21) | 1.30 (0.82, 1.78) | 1.56 (0.92, 2.21) |
| Georgia | 2.83 (1.68, 4.00) | 2.67 (1.50, 3.85) | 1.94 (0.84, 3.04) |
| Germany | -0.87 (-1.19, -0.54) | -1.44 (-1.74, -1.14) | -1.37 (-1.67, -1.07) |
| Ghana | 1.04 (0.90, 1.17) | 0.71 (0.54, 0.87) | 0.55 (0.34, 0.77) |
| Greece | 0.44 (0.03, 0.85) | 0.67 (0.07, 1.29) | 0.51 (0.01, 1.02) |
| Greenland | -0.46 (-0.89, -0.03) | -1.25 (-1.69, -0.81) | -1.36 (-1.77, -0.95) |
| Grenada | 0.98 (0.43, 1.54) | 0.29 (-0.28, 0.86) | 0.22 (-0.11, 0.54) |
| Guam | 0.87 (-0.42, 2.19) | 0.63 (-0.96, 2.25) | 1.07 (-0.17, 2.32) |
| Guatemala | 1.03 (0.63, 1.43) | 0.27 (-0.23, 0.78) | 0.42 (-0.03, 0.86) |
| Guinea | -0.00 (-0.09, 0.08) | -0.20 (-0.33, -0.06) | -0.24 (-0.40, -0.09) |
| Guinea-Bissau | 0.51 (0.40, 0.63) | 0.34 (0.21, 0.48) | 0.18 (0.07, 0.30) |
| Guyana | 1.14 (0.98, 1.31) | 0.45 (0.23, 0.66) | 0.37 (0.13, 0.61) |
| Haiti | 0.00 (-0.06, 0.06) | -0.33 (-0.38, -0.28) | -0.33 (-0.39, -0.26) |
| Honduras | 0.80 (0.28, 1.32) | 0.25 (-0.29, 0.79) | 0.22 (-0.22, 0.66) |
| Hungary | -0.79 (-1.08, -0.50) | -1.83 (-2.08, -1.58) | -1.64 (-1.82, -1.46) |
| Iceland | -0.82 (-1.47, -0.16) | -1.15 (-1.92, -0.37) | -1.06 (-1.66, -0.45) |
| India | 1.98 (1.72, 2.25) | 1.22 (0.89, 1.55) | 1.10 (0.82, 1.37) |
| Indonesia | 0.85 (0.72, 0.97) | 0.32 (0.24, 0.39) | 0.07 (-0.00, 0.15) |
| Iran (Islamic Republic of) | 1.93 (1.18, 2.69) | 0.40 (-0.33, 1.13) | 0.26 (-0.47, 0.98) |
| Iraq | 2.30 (1.91, 2.68) | 1.31 (0.81, 1.82) | 0.65 (0.31, 0.99) |
| Ireland | -0.82 (-1.50, -0.15) | -1.71 (-2.26, -1.16) | -1.81 (-2.33, -1.28) |
| Israel | -2.00 (-2.25, -1.76) | -2.58 (-2.78, -2.39) | -2.68 (-2.89, -2.47) |
| Italy | -1.56 (-1.77, -1.35) | -1.64 (-1.79, -1.48) | -1.88 (-2.11, -1.65) |
| Jamaica | 0.67 (-0.31, 1.66) | 0.68 (-0.29, 1.65) | 0.56 (-0.48, 1.60) |
| Japan | -0.03 (-0.39, 0.32) | -0.60 (-0.84, -0.36) | -0.74 (-1.00, -0.48) |
| Jordan | -1.59 (-2.12, -1.05) | -2.20 (-2.62, -1.77) | -2.43 (-2.85, -2.01) |
| Kazakhstan | -1.78 (-2.29, -1.26) | -3.28 (-4.09, -2.46) | -2.97 (-3.53, -2.41) |
| Kenya | 0.53 (0.44, 0.62) | 0.17 (0.12, 0.22) | -0.10 (-0.15, -0.05) |
| Kiribati | -0.04 (-0.15, 0.06) | -0.17 (-0.25, -0.09) | -0.24 (-0.31, -0.18) |
| Kuwait | 1.16 (-0.26, 2.59) | 0.25 (-1.11, 1.62) | -0.09 (-1.37, 1.21) |
| Kyrgyzstan | 0.14 (-0.20, 0.48) | -0.58 (-0.96, -0.21) | -0.89 (-1.15, -0.62) |
| Lao People's Democratic Republic | 0.50 (0.45, 0.55) | 0.05 (-0.00, 0.10) | -0.19 (-0.27, -0.12) |
| Latvia | -0.33 (-0.61, -0.04) | -1.47 (-1.76, -1.18) | -1.38 (-1.66, -1.10) |
| Lebanon | -0.99 (-1.49, -0.48) | -2.04 (-2.42, -1.66) | -2.01 (-2.44, -1.59) |
| Lesotho | 0.60 (0.16, 1.04) | 0.33 (-0.14, 0.80) | 0.32 (-0.12, 0.76) |
| Liberia | 0.41 (0.20, 0.62) | 0.14 (-0.07, 0.35) | 0.09 (-0.18, 0.37) |
| Libya | -0.59 (-0.79, -0.40) | -0.68 (-0.77, -0.60) | -0.54 (-0.64, -0.45) |
| Lithuania | -0.26 (-0.57, 0.04) | -1.06 (-1.38, -0.72) | -1.37 (-1.77, -0.97) |
| Luxembourg | -2.67 (-3.18, -2.16) | -3.03 (-3.39, -2.67) | -3.30 (-3.72, -2.88) |
| Madagascar | 0.68 (0.63, 0.73) | 0.52 (0.49, 0.55) | 0.39 (0.37, 0.41) |
| Malawi | 0.20 (-0.03, 0.44) | -0.06 (-0.27, 0.15) | -0.20 (-0.41, 0.01) |
| Malaysia | 1.46 (0.78, 2.15) | 0.38 (-0.34, 1.11) | 1.00 (0.37, 1.64) |
| Maldives | -0.91 (-1.22, -0.60) | -1.48 (-1.74, -1.21) | -1.73 (-2.02, -1.45) |
| Mali | 0.16 (-0.00, 0.33) | -0.02 (-0.21, 0.17) | -0.03 (-0.22, 0.17) |
| Malta | -0.42 (-0.93, 0.09) | -1.38 (-1.90, -0.86) | -1.38 (-1.84, -0.91) |
| Marshall Islands | -0.04 (-0.09, 0.01) | -0.30 (-0.34, -0.26) | -0.36 (-0.39, -0.33) |
| Mauritania | 1.68 (1.33, 2.03) | 1.10 (0.80, 1.40) | 0.88 (0.52, 1.24) |
| Mauritius | 2.96 (2.12, 3.81) | 2.38 (1.63, 3.13) | 2.45 (1.60, 3.30) |
| Mexico | 2.96 (2.67, 3.24) | 1.84 (1.48, 2.21) | 2.24 (1.91, 2.57) |
| Micronesia (Federated States of) | 0.15 (0.11, 0.19) | -0.15 (-0.17, -0.12) | -0.15 (-0.16, -0.13) |
| Monaco | -0.01 (-0.10, 0.07) | -0.39 (-0.45, -0.33) | -0.51 (-0.57, -0.44) |
| Mongolia | 1.07 (0.57, 1.57) | 0.48 (0.01, 0.95) | 0.19 (-0.28, 0.67) |
| Montenegro | 1.83 (1.35, 2.32) | 1.58 (1.15, 2.01) | 0.77 (0.31, 1.23) |
| Morocco | 2.07 (1.93, 2.20) | 1.20 (1.01, 1.38) | 0.96 (0.83, 1.09) |
| Mozambique | 0.12 (-0.18, 0.42) | -0.03 (-0.37, 0.32) | -0.17 (-0.52, 0.19) |
| Myanmar | 0.75 (0.58, 0.92) | 0.09 (-0.05, 0.24) | -0.16 (-0.33, 0.01) |
| Namibia | 0.84 (0.55, 1.13) | 0.22 (-0.06, 0.49) | 0.14 (-0.14, 0.42) |
| Nauru | 0.79 (0.72, 0.87) | 0.09 (0.04, 0.13) | -0.01 (-0.06, 0.04) |
| Nepal | 2.16 (1.89, 2.43) | 1.68 (1.38, 1.99) | 1.48 (1.19, 1.77) |
| Netherlands | -1.10 (-2.13, -0.06) | -1.07 (-2.05, -0.07) | -1.10 (-2.13, -0.06) |
| New Zealand | -0.53 (-0.93, -0.13) | -1.22 (-1.59, -0.85) | -1.28 (-1.61, -0.95) |
| Nicaragua | 0.08 (-0.61, 0.78) | -0.98 (-1.59, -0.38) | -0.80 (-1.49, -0.10) |
| Niger | 0.65 (0.42, 0.87) | 0.57 (0.34, 0.80) | 0.58 (0.32, 0.84) |
| Nigeria | 0.15 (-0.07, 0.38) | -0.14 (-0.39, 0.10) | -0.22 (-0.51, 0.07) |
| Niue | 0.45 (0.32, 0.58) | 0.04 (-0.06, 0.14) | 0.09 (-0.06, 0.25) |
| North Macedonia | -1.11 (-1.45, -0.78) | -1.42 (-1.73, -1.10) | -1.55 (-1.84, -1.25) |
| Northern Mariana Islands | 1.50 (1.20, 1.81) | 0.99 (0.43, 1.56) | 1.21 (0.88, 1.54) |
| Norway | -0.72 (-1.21, -0.22) | -0.99 (-1.46, -0.52) | -1.41 (-1.86, -0.96) |
| Oman | -0.80 (-1.71, 0.12) | -1.99 (-2.95, -1.02) | -2.64 (-3.45, -1.82) |
| Pakistan | 0.19 (0.07, 0.31) | -0.14 (-0.23, -0.04) | -0.22 (-0.30, -0.14) |
| Palau | -0.44 (-0.71, -0.18) | -0.65 (-0.97, -0.34) | -0.91 (-1.15, -0.67) |
| Palestine | -0.78 (-1.15, -0.40) | -1.93 (-2.32, -1.53) | -1.73 (-2.07, -1.39) |
| Panama | 2.54 (2.21, 2.87) | 0.70 (0.24, 1.16) | 0.93 (0.50, 1.36) |
| Papua New Guinea | 0.46 (0.28, 0.64) | 0.27 (0.12, 0.42) | 0.25 (0.06, 0.44) |
| Paraguay | 1.50 (0.95, 2.05) | 0.79 (0.20, 1.39) | 0.66 (0.16, 1.16) |
| Peru | 2.23 (1.08, 3.38) | 0.79 (-0.40, 2.00) | 0.77 (-0.46, 2.02) |
| Philippines | 0.97 (0.78, 1.17) | 0.43 (0.20, 0.66) | 0.46 (0.23, 0.70) |
| Poland | -0.13 (-0.29, 0.03) | -0.46 (-0.63, -0.29) | -0.82 (-0.98, -0.67) |
| Portugal | -1.47 (-1.68, -1.26) | -2.16 (-2.29, -2.03) | -2.32 (-2.49, -2.15) |
| Puerto Rico | -0.49 (-0.98, 0.00) | -1.64 (-2.13, -1.15) | -1.03 (-1.51, -0.54) |
| Qatar | -1.54 (-2.34, -0.74) | -3.65 (-4.93, -2.35) | -3.64 (-4.40, -2.86) |
| Republic of Korea | -0.77 (-0.99, -0.55) | -1.35 (-1.55, -1.16) | -2.05 (-2.28, -1.81) |
| Republic of Moldova | 1.05 (0.64, 1.46) | -0.37 (-0.79, 0.05) | -0.63 (-1.09, -0.16) |
| Romania | 2.11 (1.75, 2.47) | 1.21 (0.90, 1.53) | 1.10 (0.79, 1.42) |
| Russian Federation | 1.22 (0.91, 1.54) | -0.17 (-0.46, 0.13) | -0.46 (-0.77, -0.15) |
| Rwanda | 1.27 (1.10, 1.45) | 0.97 (0.81, 1.13) | 0.74 (0.59, 0.90) |
| Saint Kitts and Nevis | 0.51 (0.26, 0.77) | 0.09 (-0.19, 0.36) | 0.23 (-0.02, 0.49) |
| Saint Lucia | 1.47 (1.18, 1.76) | 1.49 (1.22, 1.77) | 1.03 (0.78, 1.28) |
| Saint Vincent and the Grenadines | 0.80 (0.43, 1.17) | 0.33 (-0.15, 0.82) | 0.56 (0.16, 0.97) |
| Samoa | 0.70 (0.57, 0.84) | 0.32 (0.14, 0.50) | 0.45 (0.23, 0.66) |
| San Marino | -3.34 (-5.63, -0.99) | -3.55 (-6.01, -1.03) | -3.47 (-5.70, -1.19) |
| Sao Tome and Principe | 1.46 (1.24, 1.69) | 0.86 (0.58, 1.14) | 0.85 (0.61, 1.08) |
| Saudi Arabia | 0.44 (0.23, 0.64) | -1.28 (-1.36, -1.20) | -1.17 (-1.27, -1.08) |
| Senegal | 1.29 (1.14, 1.45) | 1.13 (0.98, 1.29) | 0.92 (0.73, 1.12) |
| Serbia | 0.16 (-0.10, 0.43) | -0.48 (-0.72, -0.24) | -0.70 (-0.95, -0.46) |
| Seychelles | 0.02 (-0.91, 0.97) | -0.49 (-1.49, 0.52) | -0.40 (-1.29, 0.49) |
| Sierra Leone | 0.49 (0.38, 0.60) | 0.17 (0.05, 0.29) | 0.18 (0.03, 0.34) |
| Singapore | 0.90 (0.03, 1.77) | -0.18 (-1.05, 0.69) | -0.20 (-1.11, 0.72) |
| Slovakia | 0.37 (0.13, 0.62) | -0.63 (-0.81, -0.45) | -0.69 (-0.90, -0.48) |
| Slovenia | -2.09 (-2.71, -1.46) | -2.64 (-3.18, -2.09) | -3.09 (-3.67, -2.51) |
| Solomon Islands | 0.86 (0.54, 1.19) | 0.54 (0.25, 0.82) | 0.64 (0.28, 0.99) |
| Somalia | -0.29 (-0.38, -0.21) | -0.34 (-0.44, -0.25) | -0.47 (-0.52, -0.42) |
| South Africa | -0.02 (-0.35, 0.30) | -0.66 (-0.97, -0.35) | -0.73 (-1.05, -0.42) |
| South Sudan | 1.23 (1.13, 1.32) | 0.97 (0.86, 1.07) | 1.19 (1.09, 1.28) |
| Spain | -0.96 (-1.32, -0.61) | -1.59 (-1.83, -1.35) | -1.72 (-2.02, -1.43) |
| Sri Lanka | -0.03 (-0.74, 0.68) | -1.28 (-2.02, -0.54) | -1.25 (-1.84, -0.66) |
| Sudan | 1.26 (1.23, 1.29) | 0.46 (0.40, 0.51) | 0.31 (0.24, 0.38) |
| Suriname | 0.60 (-0.11, 1.32) | 0.05 (-0.69, 0.79) | 0.26 (-0.40, 0.93) |
| Sweden | -0.93 (-1.46, -0.39) | -0.92 (-1.47, -0.37) | -1.41 (-1.93, -0.89) |
| Switzerland | -1.69 (-2.15, -1.22) | -1.75 (-2.17, -1.33) | -2.20 (-2.68, -1.71) |
| Syrian Arab Republic | 0.25 (0.02, 0.49) | -0.51 (-0.57, -0.45) | -0.29 (-0.37, -0.21) |
| Taiwan (Province of China) | -0.29 (-0.67, 0.09) | -0.77 (-1.13, -0.41) | -0.70 (-1.09, -0.31) |
| Tajikistan | -1.60 (-2.00, -1.19) | -1.97 (-2.39, -1.54) | -1.80 (-2.12, -1.47) |
| Thailand | 1.71 (1.25, 2.18) | 0.58 (0.04, 1.12) | 0.87 (0.47, 1.28) |
| Timor-Leste | 1.00 (0.59, 1.41) | 0.85 (0.46, 1.24) | 0.90 (0.47, 1.33) |
| Togo | 0.73 (0.62, 0.85) | 0.42 (0.34, 0.50) | 0.28 (0.18, 0.38) |
| Tokelau | 0.50 (0.41, 0.59) | -0.22 (-0.27, -0.17) | -0.09 (-0.28, 0.10) |
| Tonga | 0.26 (0.20, 0.31) | -0.12 (-0.13, -0.11) | -0.18 (-0.20, -0.17) |
| Trinidad and Tobago | 1.53 (1.01, 2.05) | 0.60 (0.14, 1.05) | 0.91 (0.43, 1.39) |
| Tunisia | 0.83 (0.73, 0.93) | -0.42 (-0.52, -0.31) | -0.34 (-0.41, -0.26) |
| Turkey | 1.33 (1.06, 1.61) | 0.41 (0.04, 0.78) | 1.41 (0.82, 2.01) |
| Turkmenistan | 2.28 (1.73, 2.83) | 1.83 (1.31, 2.34) | -0.18 (-0.21, -0.15) |
| Tuvalu | 0.21 (0.18, 0.24) | -0.17 (-0.20, -0.14) | 0.03 (-0.23, 0.30) |
| Uganda | 0.38 (0.21, 0.54) | 0.12 (0.01, 0.22) | 0.02 (-0.08, 0.13) |
| Ukraine | -0.62 (-1.05, -0.19) | -0.77 (-1.22, -0.33) | -0.78 (-1.16, -0.39) |
| United Arab Emirates | -1.00 (-3.83, 1.92) | -1.89 (-4.77, 1.07) | -2.56 (-5.01, -0.04) |
| United Kingdom | -0.52 (-0.85, -0.19) | -0.61 (-0.90, -0.31) | -0.73 (-1.09, -0.38) |
| United Republic of Tanzania | 0.69 (0.47, 0.92) | 0.44 (0.27, 0.61) | 0.28 (0.11, 0.46) |
| United States of America | -1.12 (-1.30, -0.94) | -1.32 (-1.47, -1.17) | -0.98 (-1.12, -0.84) |
| United States Virgin Islands | -1.58 (-2.39, -0.76) | -2.20 (-3.06, -1.33) | -1.80 (-2.65, -0.95) |
| Uruguay | 0.71 (0.21, 1.21) | 0.10 (-0.35, 0.55) | 0.05 (-0.43, 0.53) |
| Uzbekistan | 3.28 (2.30, 4.26) | 2.54 (1.61, 3.49) | 2.69 (1.74, 3.64) |
| Vanuatu | 0.24 (0.18, 0.30) | 0.02 (-0.02, 0.07) | 0.13 (0.08, 0.17) |
| Venezuela (Bolivarian Republic of) | 0.74 (0.19, 1.30) | 0.69 (0.25, 1.13) | 0.86 (0.40, 1.32) |
| Viet Nam | 1.51 (1.35, 1.67) | 0.37 (0.22, 0.53) | 0.39 (0.23, 0.55) |
| Yemen | 0.12 (-0.10, 0.35) | -0.01 (-0.19, 0.17) | -0.16 (-0.39, 0.08) |
| Zambia | 1.07 (0.89, 1.25) | 0.63 (0.43, 0.83) | 0.53 (0.33, 0.72) |
| Zimbabwe | -0.18 (-0.45, 0.09) | -0.39 (-0.65, -0.12) | -0.47 (-0.74, -0.19) |

**CRC: colorectal cancer, UI: Uncertainty interval, EAPCs: Estimated annual percentage changes, DALY Disability-adjusted life-year**
