# Supplementary figures and images for: Global, regional, and national trends in colorectal cancer from 2010 to 2021: an analysis of the global burden of disease study 2021
Source: Ann Med. 2025 Aug 1;57(1):2534098. doi: 10.1080/07853890.2025.2534098 (PMC12330823; doi:10.1080/07853890.2025.2534098)

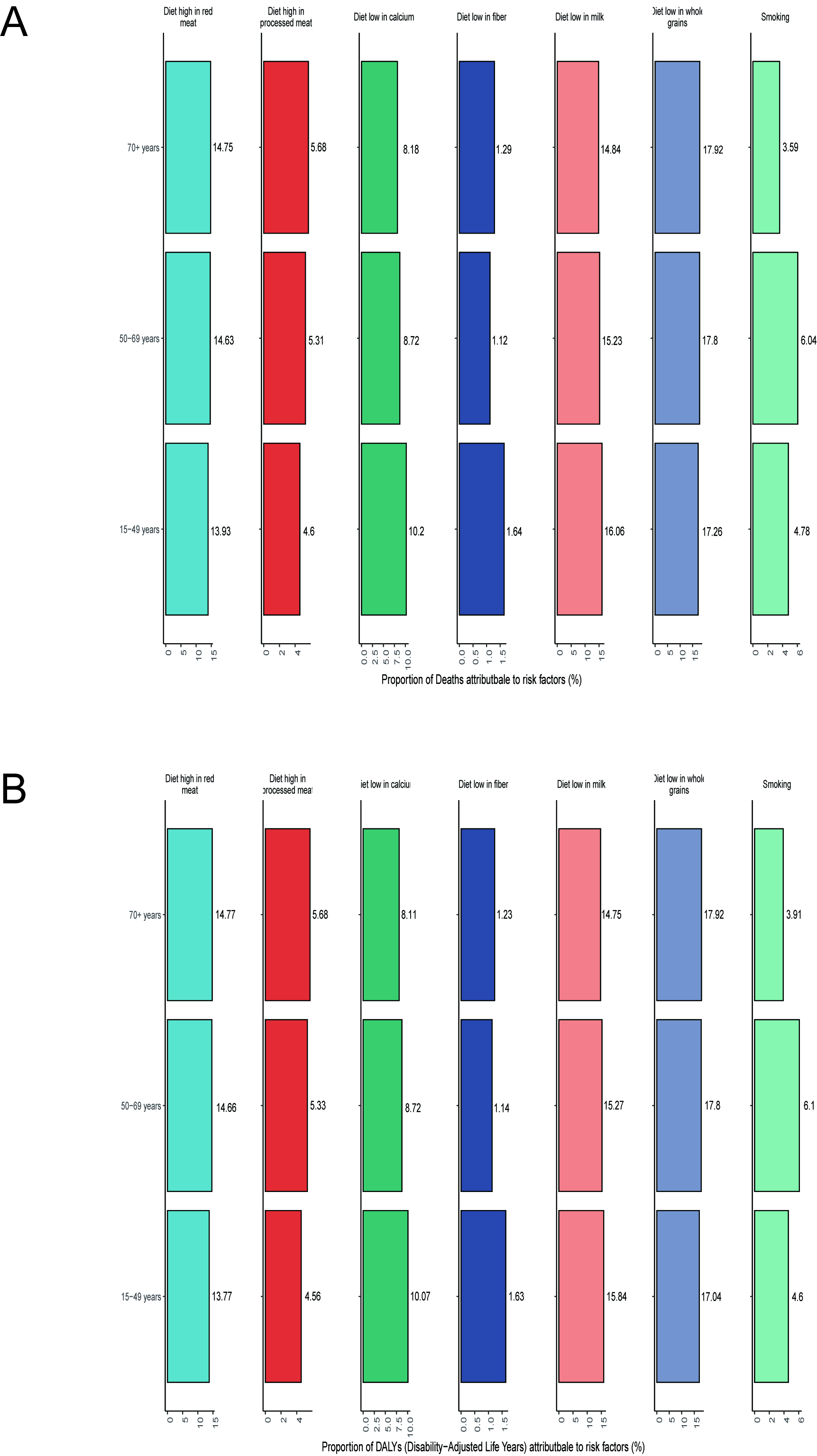

Supplement: Figure S1.tif [file IANN_A_2534098_SM1905.tif]
